# Senescence-induced p21^high^ macrophages contributed to CD8^+^ T cells-related immune hyporesponsiveness in kidney transplantation via Zfp36/IL-27 axis

**DOI:** 10.1038/s41421-025-00784-2

**Published:** 2025-04-15

**Authors:** Tingting Zhu, Qixia Shen, Lingling Shen, Yucheng Wang, Bochen Zhu, Lifeng Ma, Shi Feng, Cuili Wang, Sijing Yan, Jingyi Li, Zhimin Chen, Jingyi Zhou, Hongfeng Huang, Bingjue Li, Zhouji Shen, Qian Wang, Jianwei Wang, Wilfried Gwinner, Irina Scheffner, Song Rong, Bing Yang, Junwen Wang, Hermann Haller, Xiaoping Han, Guoji Guo, Zhinan Yin, Jin Jin, Hui-Yao Lan, Jianghua Chen, Hong Jiang

**Affiliations:** 1 Kidney Disease Center, the First Affiliated Hospital, College of Medicine, Zhejiang University, Hangzhou, Zhejiang China; 2 Institute of Nephropathy, Zhejiang University, Hangzhou, Zhejiang China; 3 Zhejiang Clinical Research Center of Kidney and Urinary System Disease, Hangzhou, Zhejiang China; 4 Department of Nephrology, Sir Run Run Shaw Hospital, Zhejiang University Medical College Affiliated, Hangzhou, Zhejiang China; 5 Bone Marrow Transplantation Center of the First Affiliated Hospital, and Center for Stem Cell and Regenerative Medicine, Zhejiang University School of Medicine, Hangzhou, Zhejiang China; 6 Ningbo Medical Center LiHuiLi Hospital, The Affiliated LiHuiLi Hospital of Ningbo University, Ningbo, Zhejiang China; 7 Guangdong Provincial Key Laboratory of Tumor Interventional Diagnosis and Treatment, Zhuhai Institute of Translational Medicine, Zhuhai People’s Hospital Affiliated with Jinan University, Jinan University, Zhuhai, Guangdong China; 8 The Biomedical Translational Research Institute, Health Science Center (School of Medicine), Jinan University, Guangzhou, Guangdong China; 9 School of Pharmaceutical Sciences, Tsinghua University, Beijing, China; 10 Department of Nephrology and Hypertension, Hannover Medical School, Hannover, Germany; 11 Zhejiang Provincial Key Laboratory for Cancer Molecular Cell Biology, Zhejiang University, Hangzhou, Zhejiang China; 12 Division of AOS & CDC, Faculty of Dentistry, The University of Hong Kong, Hong Kong SAR, China; 13 State Key Laboratory of Pharmaceutical Biotechnology, The University of Hong Kong, Hong Kong SAR, China; 14 The MOE Key Laboratory of Biosystems Homeostasis & Protection, Zhejiang Provincial Key Laboratory for Cancer Molecular Cell Biology, Life Sciences Institute, Zhejiang University, Hangzhou, Zhejiang China; 15 Department of Medicine & Therapeutics and Li Ka Shing Institute of Health Sciences, The Chinese University of Hong Kong, Hong Kong, China

**Keywords:** Immunology, Cell biology

## Abstract

Recipients’ age has emerged as a key factor that impacts on acute renal allograft rejection and graft survival. Age-related functional and structural changes in the immune system have been observed, yet the precise influence of aged immunity on kidney transplant remains unclear. In an initial retrospective analysis of clinical data gathered from two major centers in China and Germany, we found a correlation between aging and mitigated rejection outcomes in kidney recipients. To study the mechanism, we performed kidney transplantation on mice and observed attenuated allograft rejection in senescent recipients. Single-cell transcriptome analysis of allograft kidneys indicated a protective role of p21^high^ macrophages in aged mice. Supernatant collected from p21^high^ macrophage primary culture inhibited the cytotoxic function and proliferation of CD8^+^ T cells. Zfp36 is highly expressed in senescent p21^high^ macrophages. To determine its role in renal allograft rejection, we studied mice with Zfp36 conditionally deleted in macrophages (Zfp36-cKO). These mice developed exacerbated allograft rejection with enhanced IL-27 production and CD8^+^ T cell hyperactivation. Inhibition of IL-27 with neutralizing antibody or deletion of IL-27 receptor on CD8^+^ T cells reversed acute renal allograft rejection in Zfp36-cKO mice. Moreover, in vitro silencing Zfp36 with siRNA led to impaired degradation of IL-27 *p28* mRNA and a subsequent increase of IL-27 in p21^high^ macrophages. In conclusion, senescent macrophages protect renal allograft rejection by suppressing CD8^+^ T cells via a Zfp36/IL-27-dependent mechanism. These findings may provide innovative therapeutic strategies for addressing kidney allograft rejection.

## Introduction

Kidney transplantation is considered the optimal treatment for end-stage kidney disease (ESKD), offering a longer survival time and lower costs compared to dialysis^1,2^. Even older ESKD patients benefit from kidney transplantation in terms of survival and quality of life^3,4^. However, recipient age has been identified as a critical factor affecting acute rejection and graft survival^5–7^. Telomeres, the protective DNA repeats at the end of chromosomes, gradually shorten with cell division^8^, and their attrition is associated with senescence^9^. The correlation between recipient telomere length and rejection remains inconclusive due to varying sample sources and timepoints^10–12^, warranting further investigation.

Transplant kidney rejection involves complex interactions between innate and adaptive immunity, and aging leads to significant changes in the immune system, making individuals more susceptible to infections, autoimmunity, and cancer^13,14^. However, studies on the functional impact of age-related immune alterations on immune response to the transplanted organ are still limited. Idiopathic pulmonary fibrosis lung transplant recipients with short telomere have premature “aging” of their circulating T cell compartment posttransplantation, with increased granzyme B positivity of both CD8^+^ and CD4^+^ T cells, upregulation of the exhaustion marker (CD57) and chemotactic protein (CCR5), and enhanced T cell receptor clonal expansion^15^. Compromise of CD8^+^ T cell functions including naive–memory imbalance was also observed in recipients older than 65 years, but whether it is the cause of the attenuation rejection in senescent recipients is unknown^16^. Understanding how the immune system changes with senescence could aid clinicians in applying a more personalized immunosuppression approach for older recipients, preventing inappropriate immunosuppression^17^. Interestingly, not all immune processes are equally affected by aging, suggesting heterogeneous impairments in different immune cell populations during senescence^18^. To explore the precise mechanisms of age-dependent alterations impacting rejection and overall graft survival, we conducted single-cell RNA sequencing (scRNA-seq) analysis of transplanted kidneys in young and senescent recipients.

Our study revealed a correlation between recipient senescence and kidney transplant rejection in our cohorts. Moreover, we identified that increased expression of Zfp36 in senescent p21^high^ macrophages led to reduced IL-27 production by accelerating IL-27 *p28* mRNA decay. This impairment in IL-27 led to dysfunction of CD8^+^ T cells, ultimately resulting in attenuated rejection.

## Results

### Senescent recipients exhibit a lower rejection rate and a better graft survival after kidney transplantation

**Fig. 1:**
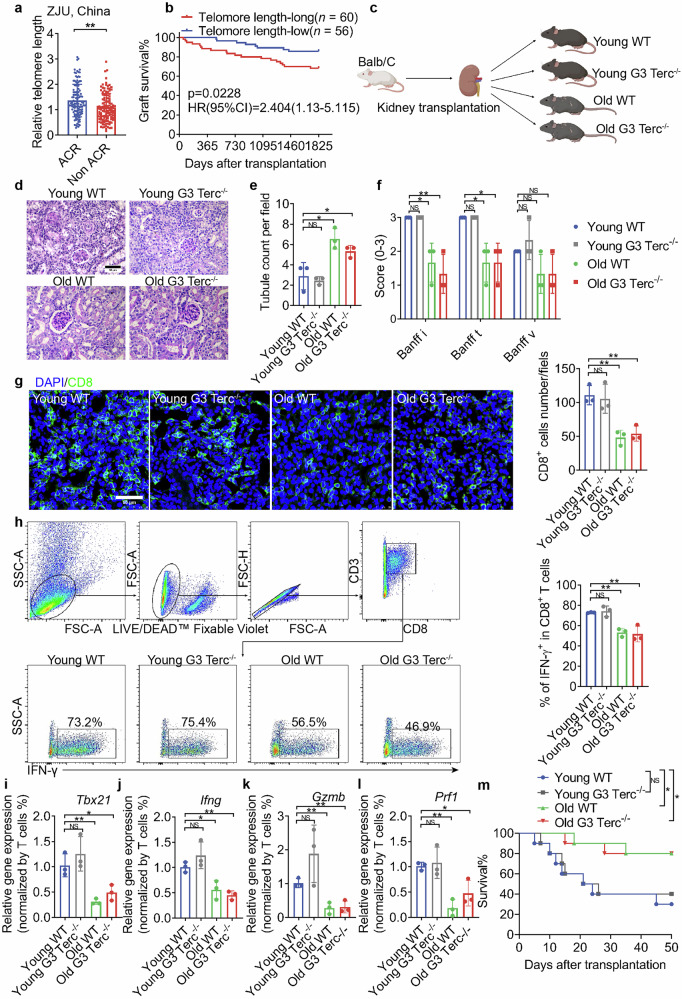
Senescent recipients showed reduced rejection following kidney transplantation. **a** Relative telomere length of PBMCs collected before surgery in ACR and Non-ACR patients from ZJU, China using T/S methods, *n* = 119/group. **b** Kaplan-Meier curves of allografts stratified by long and short telomere, *n* = 60 and *n* = 56, respectively. **c** Schematic diagram of the animal experimental design. **d** Representative image of PAS staining of Young WT, Young G3 Terc^*−/−*^, Old WT and Old G3 Terc^*−/−*^ mouse allografts. Scale bar, 50 μm. **e** Quantitative analysis of healthy tubule count per field, *n* = 3/group. **f** Quantitative analysis of i, t and v scores according to Banff 2019, *n* = 3/group. **g** Representative image of CD8^+^ T cells (CD8, green) immunofluorescent staining of allografts. Scale bar, 50 μm. Quantitative analysis of CD8^+^ T cell numbers per field is shown on the right side, *n* = 3/group. **h** Flow cytometric gating of lymphocytes, living cells, single cells, and CD8^+^ T cells. Representative flow panels of IFN-γ^+^CD8^+^ T cells from allografts of three groups of mice are shown. Quantitative analysis of the IFN-γ^+^CD8^+^ T ratios are shown on the right side, *n* = 3/group. **i** Expression level of *Tbx21* normalized for the numbers of T cells in allografts analyzed using qPCR, *n* = 3/group. **j** Expression level of *Ifng* normalized for the numbers of T cells in allografts analyzed using qPCR, *n* = 3/group. **k** Expression level of *Gzmb* normalized for the numbers of T cells in allografts analyzed using qPCR, *n* = 3/group. **l** Expression level of *Prf1* normalized for the numbers of T cells in allografts analyzed using qPCR, *n* = 3/group. Data are presented as mean ± SD. **m** Kaplan-Meier curves of Young WT, Young G3 Terc^*−/−*^, Old WT and Old G3 Terc^*−/−*^ mice treated with Tacrolimus 1 mg/kg/day after transplantation, *n* = 10/group. Statistical analysis was performed using two-tailed Student’s *t*-test (**a**, **d**–**j**) or Log-Rank test (**b**). *P < 0.05, **P < 0.01, ***P < 0.001, ****P < 0.0001. ACR, Acute cellular rejection. Non-ACR, Non-acute cellular rejection.

Patients diagnosed with ESKD from Zhejiang University (ZJU), China who received kidney transplantation between 2011.1.1 and 2021.12.31 were included in our study. All patients were followed up after surgery. After inclusion and exclusion criteria were established (Supplementary Fig. S1a), a total of 119 patients exhibiting acute cellular rejection (ACR) within one year after transplantation and 119 matched patients without rejection (Non-ACR) were recruited into this study. Demographics and baseline characteristics of recipients and donors, donor-recipient matching information and immunosuppression therapy were shown in Supplementary Table S1. Banff type of rejection was provided in Supplementary Table S2. Telomere to single copy gene (T/S) ratio methods were utilized to determine the relative telomere length of peripheral blood mononuclear cells (PBMCs) collected prior to surgery in ACR and Non-ACR patients. It was found that the ACR group had longer telomeres than the non-ACR group (Fig. 1a). Other cohort from Medizinische Hochschule Hannover (MHH), Germany showed similar results (Supplementary Fig. S1b). To further explore the link between telomere length and graft survival, we investigated 116 patients who underwent surgery before December 2017 by following up over the course of five years in ZJU cohort. Importantly, graft survival was better in patients exhibiting shorter telomeres (Fig. 1b).

To clarify this phenomenon, the kidneys of BALB/c (8 weeks) mice were transplanted into fully MHC-mismatched Young wild-type (WT) (8 weeks), Young G3 Terc^*−/−*^ (the third generation of RNA component of telomerase deficient mice, 8 weeks), Old WT (18 months), and Old G3 Terc^*−/−*^ (12 months) recipients (Fig. 1c). Absence of Terc leads to telomere shortening progressively during successive generations of Terc-deficient mice^19^. Thus, G3 Terc^*−/−*^ mice develop the senescence phenotype earlier than WT mice after birth and were employed in this study. Allografts from the three groups were collected 7 days after surgery. As the surgery did not remove the recipient’s native kidneys, even if severe rejection occurred after surgery, the serum creatinine levels could still be maintained within the normal range due to the compensatory effect of the kidney present in situ^20^. Periodic Acid-Schiff (PAS) staining was conducted (Fig. 1d), and the number of healthy tubules was determined to indirectly show the graft function (Fig. 1e). According to the Banff 2019 T-cell-mediated rejection (TCMR) diagnostic criteria^21^, renal interstitial inflammation (i), tubulitis (t), and intrarenal arteritis (v) were scored. The results showed that the i and t scores in the Young WT and Young G3 Terc^*−/−*^ group were significantly higher than those in Old WT and Old G3 Terc^*−/−*^ group (Fig. 1f). Immunofluorescent assessment of allografts demonstrated lower accumulation of CD8^+^ T in senescent mice (Fig. 1g), and the IFN-γ^+^CD8^+^ T ratio was also decreased (Fig. 1h) in Old WT and Old G3 Terc^*−/−*^ group. Moreover, the expression of a crucial transcription factor for CD8^+^ T cell activation (*Tbx21*) and effector molecules (*Ifng*, *Gzmb* and *Prf1*) was substantially reduced in the allografts of senescent mice (Fig. 1i–l). In addition, no difference among the four groups was observed with respect to the proportion of Th1, Th2, Th17, and Treg (Supplementary Fig. S2b–e). In consistent with previous study^22^, our data showed a decrease in Naive CD8^+^ T in lymphoid organs of senescent mice, although it was comparable in allografts (Supplementary Fig. S2a, f and l). No difference among the four groups was observed in the proportion of Th1, Th2, Th17, Treg and IFN-γ^+^CD8^+^ T in spleen (Supplementary Fig. S2g–k). In accordance with 7 days results, Old WT and Old G3 Terc^*−/−*^ exhibited a better survival than Young WT, Young G3 Terc^*−/−*^ treated with Tacrolimus 1 mg/kg/day, which is the first-line immunosuppressant which consists of the footstone as immunosuppressive regimens in kidney transplantation (Fig. 1m). These data demonstrated that senescent recipients developed lower graft rejection after kidney transplantation.

### scRNA-seq reveals an impaired CD8^+^ T cell-mediated renal allograft rejection in senescent mice and in G3 Terc-deficient mice

**Fig. 2:**
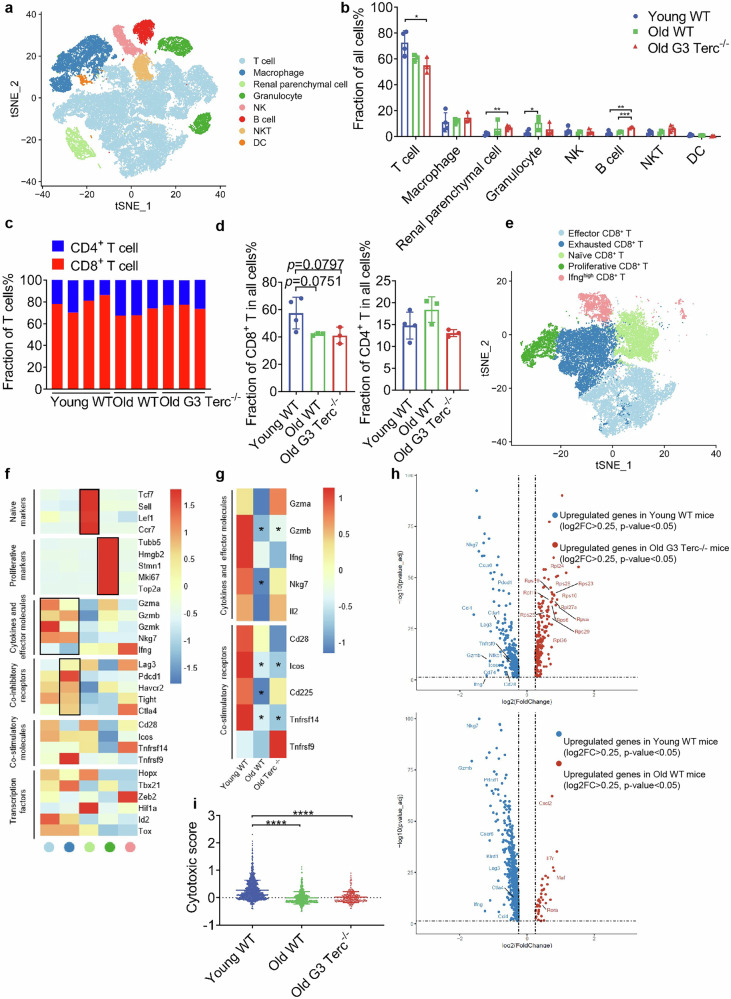
Impaired cytotoxic function of graft-infiltrated CD8^+^ T cells in senescent mice. **a** *t*-SNE plot displaying 8 cell clusters from Young WT (*n* = 4), Old WT (*n* = 3), and Old G3 Terc^*−/−*^ (*n* = 3) mouse allograft. **b** Frequencies of clusters in the three groups. **c** Fraction of CD4^+^ T and CD8^+^ T in individuals. **d** Fraction of CD4^+^ T and CD8^+^ T cells in the three groups. **e** Identification of 5 subclusters of CD8^+^ T cells. **f** Normalized mean expression of selected CD8^+^ T cell function-associated genes in each cell cluster. Black boxes highlight the prominent patterns defining known CD8^+^ T cell subtypes. **g** Normalized mean expression of the effector CD8^+^ T subcluster in the three groups, with an asterisk indicating a significant difference from Young WT. **h** Volcano plot of the effector CD8^+^ T subcluster showing the log2 fold change (*x*-axis) and –log10(*P* value_adj) (*y*-axis) of the differential analysis. Each dot represents a gene. The dashed horizontal black line represents a *P* value_adj of 0.05. The dashed vertical black line represents a |log2 fold change| of 0.25. Significant genes (*P* value_adj < 0.05, |log2 fold change | > 0.25) are shown, with their names displayed alongside the dots. **i** Cytotoxic score of the effector CD8^+^ T cluster in three groups. Data are presented as mean ± SD. Statistical analysis was performed using two-tailed Student’s *t*-test (**b**, **d**, **i**). *P < 0.05, **P < 0.01, ***P < 0.001, ****P < 0.0001.

In order to examine the intrinsic causes of attenuated rejection in senescent mice, we performed single-cell transcriptome analysis of the allograft in Young WT (*n* = 4), Old WT (*n* = 3), and Old G3 Terc^*−/−*^ (*n* = 3) mice. A total of 54,484 cells met our quality control standards. We identified 8 clusters, including T cells, macrophages, renal parenchymal cells, granulocytes, NKs, B cells, NKTs, and DCs (Fig. 2a; Supplementary Fig. S3a). Cell types corresponded to classical marker genes from CellMarker and the Mouse Cell Atlas (MCA) databases and published literature^23,24^ (Supplementary Fig. S3b, c). The T cell cluster accounted for over 40% across groups or in individuals (Supplementary Fig. S3d, e). Furthermore, the relative proportions of these cell types in senescent mice indicated a reduction in T cells and an increase in renal parenchymal cells, granulocytes, and B cells compared to young mice (Fig. 2b). Next, we examined the proportion of CD4^+^ and CD8^+^ T cells and uncovered that CD8^+^ T cells accounted for 60%–80% of all T cells, operating as a major T cell component in TCMR (Fig. 2c). The proportion of CD8^+^ T cells in the Young WT group was higher than the Old WT and Old G3 Terc^*−/−*^ groups (*P* = 0.0751; *P* = 0.0797). However, there was no difference in CD4^+^ T cells between groups (Fig. 2d). These findings indicated that the decrease in the percentage of T cells in senescent recipients was primarily due to the reduction of CD8^+^ T cells.

Therefore, we performed reclustering of CD8^+^ T cells. Five subclusters of CD8^+^ T cells were identified (Fig. 2e), and the signature gene expression levels and known functional markers indicated the presence of subclusters of CD8^+^ T, including Effector CD8^+^ T (Gzma, Gzbm, Gzmk, Nkg7, and Ifng), Exhausted CD8^+^ T (Lag3, Pdcd1, Havcr2, Tight, and Ctla4), Naive CD8^+^ T (Tcf7, Sell, Lef1 and Ccr7), Proliferative CD8^+^ T (Tubb5, Hmgb2, Stmn1, Mki67, and Top2a), and Ignfhigh CD8^+^ T (especially high expression of Ifng) (Fig. 2f). Based on the critical role of effector CD8^+^ T cells in transplantation immunity and the difference in the IFN-γ^+^CD8^+^ T ratio between young and senescent recipients (Fig. 1h), we analyzed cytokines and effector molecules, as well as co-stimulatory receptors in three groups of the CD8^+^ T subcluster. The data demonstrated a reduction in the expression level for signature genes in the two senescent groups compared with Young WT (Fig. 2g). The volcano plot for the effector CD8^+^ T subcluster also demonstrated that cytotoxic functioning was impaired in senescent mice (Fig. 2h). Moreover, the effector CD8^+^ T subcluster of Old WT and Old G3 Terc^*−/−*^ mice were determined to have a lower cytotoxic score than that in Young WT (Fig. 2i). Similar results were obtained in the Ifnghigh CD8^+^ T subcluster (Supplementary Fig. S4a-b). These data demonstrated that the cytotoxic functions of graft-infiltrated CD8^+^ T cells were impaired in senescent mice.

To determine the contribution of CD4^+^ T cells in senescent recipient kidney transplant rejection, we also performed reclustering of CD4^+^ T cells, identifying five subclusters (Supplementary Fig. S4c). The expression of signature genes and known functional markers indicated the presence of clusters of CD4^+^ T, including Exhausted CD4^+^ T (Lag3, Pdcd1, Havcr2, and Ctla4), Treg (Il2ra, Foxp3, and Ikzf2), Naive CD4^+^ T (Tcf7, Sell, Lef1 and Ccr7), Activated CD4^+^ T/Th1 (Il2, Gzma, Gzmb, Gzmk, Ifng, and Nkg7), and Th17 (Il17a, Ccr6, Ccr4, Rora and Rorc) (Supplementary Fig. S4d). The proportions of each subcluster across the three groups were consistent (Supplementary Fig. S4e). Considering the altered cytotoxic functioning of CD8^+^ T cells, we analyzed differentially expressed genes in the activated/Th1 cell subcluster, and minimal differential genes were identified in the Old G3 Terc^*−/−*^ group and the Old WT group compared to the Young WT group (Supplementary Fig. S4f). These results confirmed that CD4^+^ T cells were not the primary effector cells for attenuating rejection in senescent mice.

### p21^high^ macrophages are increased in senescent mice and function to inhibit CD8^+^ T cell activation during renal allograft rejection

**Fig. 3:**
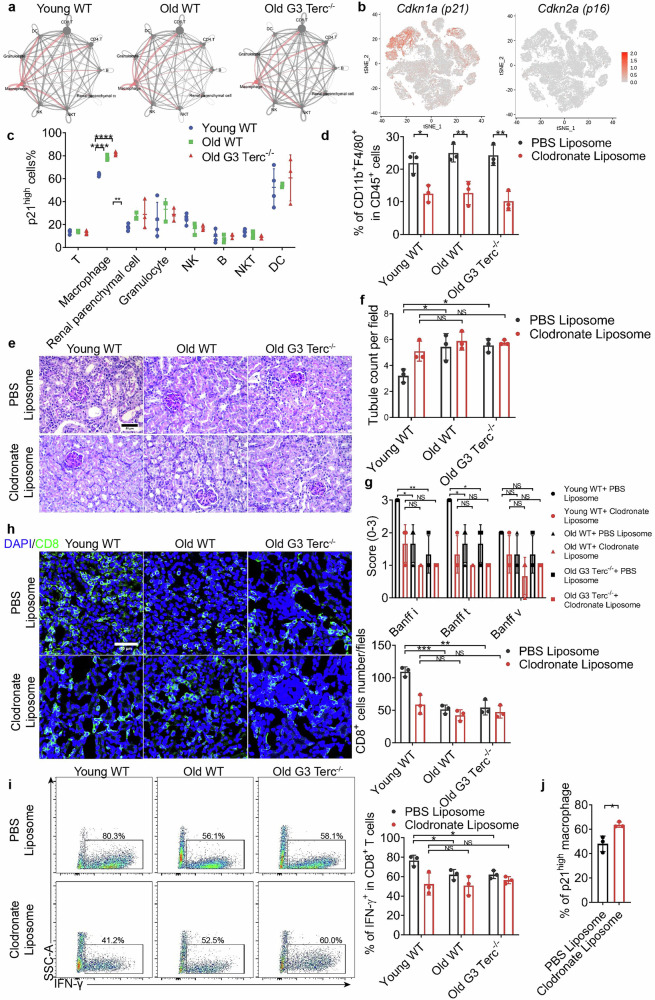
Accumulation of p21^high^ macrophages in senescent mice contributes to reduced rejection following kidney transplantation. **a** Interaction net count plot of allograft cells revealed by CellChat, with the thickness of the lines representing the number of interactions between the two cell types. **b** Senescence marker genes (*Cdkn1a* and *Cdkn2a*) expression projected on *t*-SNE plots (cell type refer to Fig. 2a), with red denoting high expression and gray denoting low expression. **c** Proportion of p21^high^ cells in all cell types. **d** Graft-infiltrated macrophage in Young WT, Old WT and Old G3 Terc^*−/−*^ mouse allografts treated with PBS Liposome or Clodronate Liposome. **e** Representative image of PAS of Young WT, Old WT and Old G3 Terc^*−/−*^ mouse allografts treated with PBS Liposome or Clodronate Liposome. Scale bar, 50 μm. **f** Quantitative analysis of healthy tubule count per field, *n* = 3/group. **g** Quantitative analysis of i, t and v scores according to Banff 2019, *n* = 3/group. **h** Representative image of CD8^+^ T cells (CD8, green) immunofluorescent staining of allografts. Scale bar, 50 μm. Quantitative analysis of CD8^+^ T cell numbers per field is shown on the right side, *n* = 3/group. **i** Flow cytometric gating of lymphocytes, living cells, single cells, and CD8^+^ T cells. Representative flow panels of IFN-γ^+^CD8^+^ T cells from allografts are shown. Quantitative analysis of the IFN-γ^+^CD8^+^ T ratios are shown on the right side, *n* = 3/group. **j** Proportion of p21^high^ macrophage in all macrophages of Young WT mouse allografts treated with PBS Liposome or Clodronate Liposome, *n* = 3/group. Data are presented as mean ± SD. Statistical analysis was performed using two-tailed Student’s *t*-test (**c**–**h**). *P < 0.05, **P < 0.01, ***P < 0.001, ****P < 0.0001, NS, not significant.

To clarify the cytotoxic dysfunction of CD8^+^ T cells in senescent individuals, we initially utilized CellChat to evaluate the major signaling inputs and outputs across all cell clusters. We first determined that communication between CD8^+^ T cells, macrophages and DCs in senescent mice, particularly in the Old G3 Terc^*−/−*^ group (Fig. 3a). Next, we investigated the expression of p21 and p16, two major regulators and markers of senescence^25^. p21 was predominantly expressed in macrophages and DCs, with moderate to low expression in granulocytes and some T cells, and very low expression in other cell types. In contrast, p16 was low-expressed across all cell clusters (Fig. 3b). Therefore, p21 was selected as the marker to identify senescent cells. The p21^high^ macrophages were more abundant in allografts of senescent mice (Fig. 3c). In vitro, bone marrow-derived macrophages (BMDMs) from Old WT and Old G3 Terc^*−/−*^ mice possessed shorter telomere than those from Young WT (Supplementary Fig. S5a), more SA-β-Gal^+^ cells and elevated expression of p21 (Supplementary Fig. S5b, c). In another irradiation-induced senescence model (Supplementary Fig. S6a), 10 Gy of irradiation caused a substantial increase in the proportion of SA-β-Gal^+^ cells and the expression of p21 (Supplementary Fig. S6b, c). These data indicated that the dysfunction of CD8^+^ T cells may be due to senescent macrophages.

To evaluate the functional role of macrophage in allograft rejection in senescent recipients, we performed macrophage depletion in vivo. Surprisingly, when macrophages were partially depleted by Liposome (Fig. 3d), healthy tubules were retained, Banff i and t scores were lower, while accumulation of CD8^+^ T and IFN-γ^+^CD8^+^ T ratio in allografts were comparable in young and senescent mice 7 days after surgery (Fig. 3e–i), indicating a role for macrophages in renal allograft rejection in senescent mice. Interestingly, the changes in allograft rejection were only observed in young mice, thus we further checked the depletion efficiency of p21^low^ and p21^high^ macrophages. Our data showed that the proportion of p21^high^ macrophages was increased after Clodronate liposomes treatment (Fig. 3j), which indicated that macrophage phagocytic ability decreases with aging and liposomes may preferentially target p21^low^ macrophages. In view of comparable depletion efficiency (Fig. 3d), it could be speculated that p21^low^ macrophages may play a more important role in graft rejection.

**Fig. 4:**
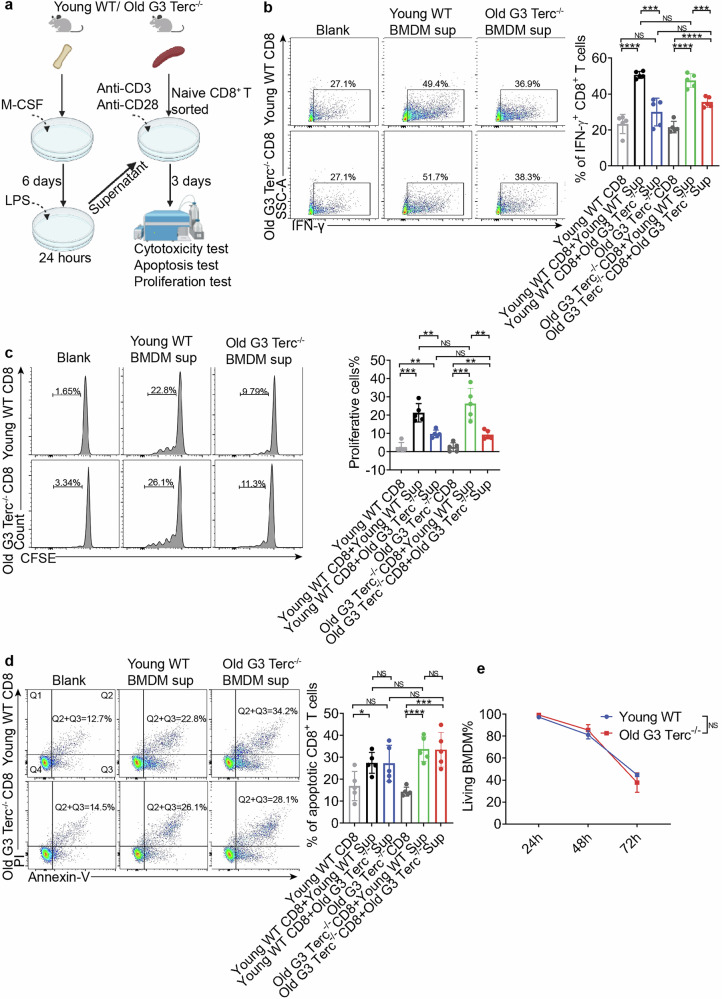
Senescent macrophages contribute to CD8^+^ T cell dysfunction. **a** Flowchart of naive CD8^+^ T cells cultured with BMDM supernatant. Young WT and Old G3 Terc^*−/−*^ BMDMs were stimulated by LPS (1 μg/mL) for 24 h, and supernatants were collected. Purified naive Young WT and Old G3 Terc^*−/−*^ CD8^+^ T cells were cultured with the supernatants (1:1) in the presence of plate-coated α-CD3 Abs and α-CD28 Abs for 3 days. Cytotoxicity, apoptosis, and proliferation tests were detected by FACS. **b** Representative flow panels of IFN-γ^+^CD8^+^ T cells. Quantitative analysis of the IFN-γ^+^CD8^+^ T ratios is shown on the right side, *n* = 3/group. **c** Representative flow panels of CFSE-labeled CD8^+^ T cells. Quantitative analysis of the proliferative CD8^+^ T cells is shown on the right side, *n* = 5/group. **d** Representative flow panels of apoptotic CD8^+^ T cells. Quantitative analysis of the apoptotic CD8^+^ T cells is shown on the right side, *n* = 5/group. **e** Percentage of living BMDM 24 h, 48 h and 72 h after LPS stimulation, *n* = 3/group. Data are presented as mean ± SD. Statistical analysis was performed using two-tailed Student’s *t*-test (**b**–**d**) or Two-Way ANOVA (**e**). **P < 0.01, ***P < 0.001, ****P < 0.0001, NS, not significant. BMDM, bone marrow-derived macrophages.

Unlike the major effect of DCs in directing the stimulation of the immune response against the allograft, there remains little direct evidence of macrophages acting specifically as antigen presenting cells. While, activated macrophages secrete a large number of pro-inflammatory or anti-inflammatory cytokines, which are crucial in immunomodulation^26^. To further determine if the dysfunction of CD8^+^ T cells in senescent mice is intrinsic or is regulated by senescent macrophages, we purified naive CD8^+^ T cells from Young WT and Old G3 Terc^*−/−*^ mice. These cells were cultured with supernatants collected from activated Young WT and Old G3 Terc^*−/−*^ BMDM supernatants in vitro and measured cytotoxic function, proliferative capacity, and apoptosis (Fig. 4a). Macrophage supernatant promoted activation of Naive CD8^+^ T cells. Interestingly, supernatants from Old G3 Terc^*−/−*^ BMDM induced attenuated IFN-γ^+^CD8^+^ T cells from Young WT and Old G3 Terc^*−/−*^ compared to the supernatant from Young WT BMDM. Notably, Young WT and Old G3 Terc^*−/−*^ CD8^+^ T cells exhibited similar IFN-γ^+^CD8^+^ T percentages after three days when cultured using the same BMDM supernatants (Fig. 4b). In addition, macrophage supernatant from Old G3 Terc^*−/−*^ BMDM also showed an attenuated promotion effect on CD8^+^ T cell proliferation when compared to the Young WT BMDM supernatant, whereas, the proliferative activity was comparable between Young WT and Old G3 Terc^*−/−*^ CD8^+^ T cells (Fig. 4c). Interestingly, though macrophage supernatant promoted apoptosis of CD8^+^ T cells, no difference was observed in apoptosis between groups (Fig. 4d). Senescent BMDM were not short-lived as to explain the above phenomenon (Fig. 4e). These results suggested that reduced cytotoxicity and proliferative capacity of CD8^+^ T cells is not an intrinsic defect in the senescent mice, but instead driven by p21^high^ macrophages accumulated in senescent mice.

### Senescent macrophages protect against CD8^+^ T cell-mediated renal allograft rejection via Zfp36-dependent mechanism

**Fig. 5:**
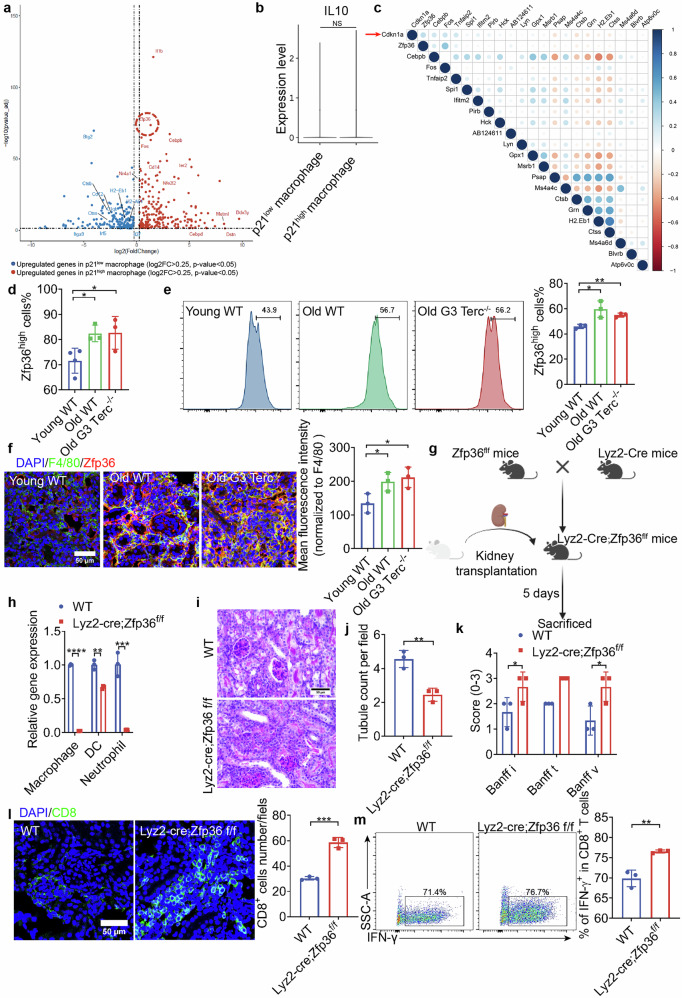
Zfp36 was upregulated in macrophages of senescent mice, and Lyz2-cre;Zfp36^f/f^ mice exhibited aggravated acute rejection. **a** Volcano plot of p21^high^ and p21^low^ macrophages showing the log2 fold change (*x*-axis) and –log10(*P* value_adj) (*y*-axis) of gene expression. Each dot represents a gene. The dashed horizontal black line represents a *P* value_adj of 0.05. The dashed vertical black line represents a |log2 fold change| of 0.25. Significant genes (*P* value_adj < 0.05, |log2 fold change | > 0.25) are shown, with their names displayed alongside the dots. **b** Expression level of IL-10 in p21^low^ and p21^high^ macrophages from scRNA sequencing. **c** Pearson Correlation Analysis of key genes, where blue represents positive correlation, and red represents negative correlation. The darker the color and bigger the dot size, the stronger the correlation. **d** Proportion of Zfp36high cells in Young WT (*n* = 4), Old WT (*n* = 3), and Old G3 Terc^*−/−*^ (*n* = 3) mouse allograft analyzed in scRNA-seq. **e** Representative flow panels of Zfp36 expression in macrophages. Quantitative analysis of the proportion of Zfp36high macrophages is shown on the right side, *n* = 3/group. **f** Represe*n*tative image of Zfp36 in macrophages (F4/80, green and Zfp36, red) immunofluorescent staining of allografts; semiquantitative analysis of the Zfp36 expression normalized to F4/80 is shown on the right side, *n* = 3/group. Scale bar, 50 μm. Quantitative analysis of CD8^+^ T cells number per field is shown on the right side, *n* = 3/group. **g** Schematic diagram of experimental design using Lyz2-cre;Zfp36f/f mice. **h** Expression level of Zfp36 of macrophage, DC and neutrophil detected by qRT-PCR in WT and Lyz2-cre;Zfp36f/f mice, *n* = 3/group. **i** Represe*n*tative image of PAS staining of allografts. Scale bar, 50 μm. **j** Quantitative analysis of healthy tubule count per field, *n* = 3/group. **k** Quantitative analysis of i, t and v scores according to Banff 2019, *n* = 3/group. **l** Represe*n*tative image of CD8^+^ T cells (CD8, green) immunofluorescent staining of allografts. Scale bar, 50 μm. Quantitative analysis of CD8^+^ T cells number per field is shown on the right side, *n* = 3/group. **m** Represe*n*tative flow panels of IFN-γ^+^CD8^+^ T cells from allografts are shown. Quantitative analysis of the IFN-γ^+^CD8^+^ T ratios is shown on the right side, *n* = 3/group. Data are presented as mean ± SD. Statistical analysis was performed using two-tailed Student’s *t*-test (**d**, **e**, **i**–**k**). *P < 0.05, **P < 0.01, ***P < 0.001.

To identify gene expression patterns in aging macrophages, differential gene analysis of p21^high^ and p21^low^ macrophages was performed. A total of 210 genes, including *Zfp36*, *Cebpb*, *Cebpd*, *and Ier2*, were upregulated in senescent macrophages, while 173 genes, such as *Btg2, C1qb, H2-Ab*, and *IL-27*, were downregulated (Fig. 5a). *IL-10* expression was similar in p21^low^ and p21^high^ macrophage (Fig. 5b). We screened candidate genes using the following criteria: significantly increased expression in p21^high^ macrophages (p_val_adj < 0.05, |logfc.threshold | > 1), and the proportion of cells expressing the gene in p21^high^ macrophages was over 80%. *Zfp36* expression was most relevant to *p21* (Fig. 5c). The proportion of Zfp36high macrophages was increased in Old WT and Old G3 Terc^*−/−*^ compared to the Young WT allograft (Fig. 5d). FACS and immunofluorescence results further confirmed the findings based on bioinformatics (Fig. 5e, f). In vitro, *Zfp36* was upregulated in BMDMs from Old WT and Old G3 Terc^*−/−*^ or cells treated with irradiation (Supplementary Figs. S5d and S6d).

To investigate whether overexpression of Zfp36 in macrophages is linked to CD8^+^ T cell dysfunction as well as attenuated rejection after kidney transplantation, we established a TCMR model in macrophage Zfp36-conditional knockout mice (Fig. 5g). Zfp36 was significantly reduced in macrophage and neutrophil, rather than DC in Lyz2-cre;Zfp36f/f mice (Fig. 5h). Notably, neutrophil is recognized to mediate early graft injury^27^. Our data also showed that neutrophil depletion had little effect on IFN-γ^+^CD8^+^ T ratio in allografts (Supplementary Fig. S7); thus Lyz2-cre;Zfp36f/f mice can be used for the following macrophage-specific knockout studies. PAS staining revealed that the graft in Lyz2-cre;Zfp36f/f mice exhibited a higher proportion of unhealthy renal tubules and higher Banff i, t, v scores (Fig. 5i–k). Additionally, there was a notable increase in CD8^+^ T cell infiltration in Lyz2-cre;Zfp36f/f mice compared to WT mice (Fig. 5l), and the ratio of IFN-γ^+^CD8^+^ T cells was also elevated (Fig. 5m). These observations indicated that the deficiency of Zfp36 in macrophages resulted in exacerbated acute rejection.

### Overexpression of Zfp36 in senescent macrophages promotes IL-27 *p28* mRNA degradation and inhibits IL-27 signaling

**Fig. 6:**
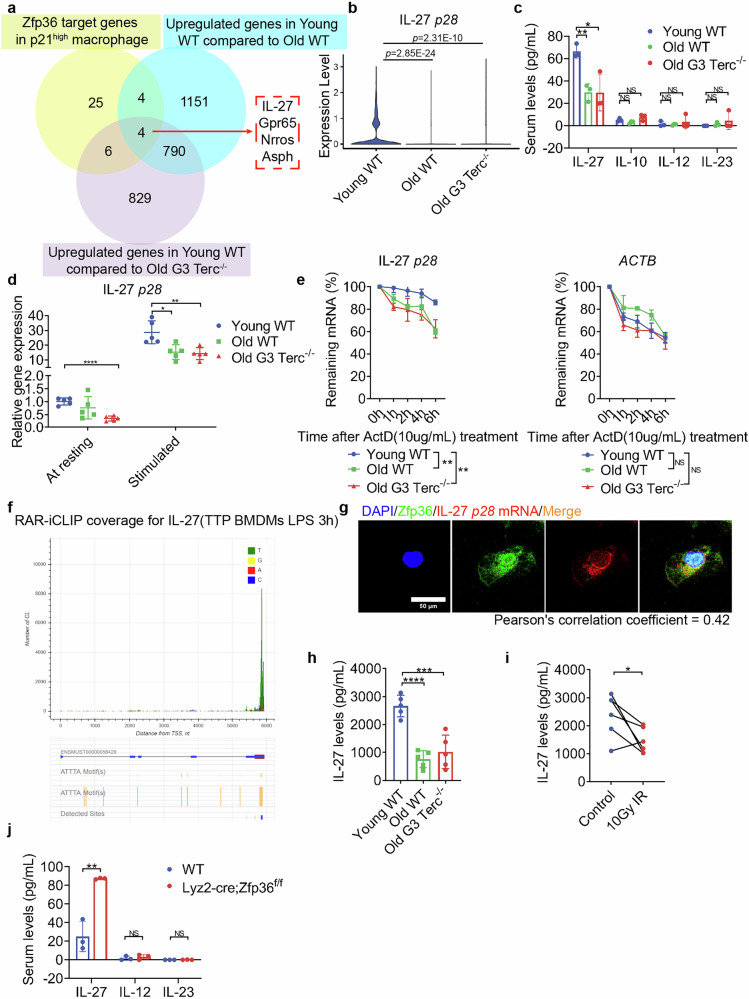
Zfp36 overexpression in senescent mouse macrophages accelerated IL-27 *p28* mRNA degradation and reduced IL-27 release. **a** Venn diagram showing the overlap of Zfp36 target genes in p21^high^ macrophages, upregulated genes in the Young WT group compared to Old WT, and upregulated genes in the Young WT group compared to Old G3 Terc^*−/−*^. **b** Expression level of IL-27 *p28* in macrophages of Young WT, Old WT, and Old G3 Terc^*−/−*^ mouse allografts. **c** IL-27, IL-10, IL-12 and IL-23 levels in mouse serum 7 days after surgery by ELISA, *n* = 3/group. **d** Expression level of BMDM IL-27 *p28* at rest or stimulated by LPS analyzed using qPCR, *n* = 5/group. **e** IL-27 *p28* and *ACTB* mRNA degradation analyzed using qPCR after blocking RNA de novo synthesis using ActD, *n* = 5/group. **f** PAR-iCLIP coverage for IL-27. **g** Confocal microscopy analysis of the colocalization of Zfp36 (green) and IL-27 *p28* mRNA (red). Scale bar, 50 μm. **h** IL-27 levels in BMDM supernatants of Young WT, Old WT, and Old G3 Terc^*−/−*^ mice by ELISA, *n* = 5/group. **i** IL-27 levels in supernatants of BMDM with or without irradiation detected by ELISA, *n* = 5/group. **j** IL-27, IL12 and IL23 levels in WT and Lyz2-cre;Zfp36f/f mice, *n* = 3/group. Data are presented as mean ± SD. Statistical analysis was performed using two-tailed Student’s *t*-test (**b**–**d**, **g**–**i**) or Two-Way ANOVA (e). *P < 0.05, **P < 0.01, ***P < 0.001, ****P < 0.0001, NS, not significant. BMDM, bone marrow-derived macrophages.

Zfp36 is a member of the Zfp36 family and is characterized by the presence of one or more CCCH-type zinc finger domains, which contain three cysteines (C) and one histidine (H) residue. The Zfp36 family proteins bind to adenylate-uridylate-rich elements (AREs) located in the 3’-untranslated region (3’ UTR) of target mRNAs, resulting in the degradation of these mRNAs^28^. Zfp36 plays a significant role in regulating immune responses and inflammatory diseases by inhibiting the production of various inflammatory cytokines, including TNF-α, IL-17, and IL-23^29,30^. Therefore, we attempted to uncover the target mRNA of Zfp36 using the following criteria: (1) downregulation in p21^high^ macrophage with AREs in the 3’ UTR^31^; (2) upregulation in the Young WT group compared to Old WT; (3) upregulation in the Young WT group compared to Old G3 Terc^*−/−*^ (Fig. 6a). Among the four overlapping genes, Gpr65 is predominantly expressed in T cells^32^, Nrros is linked to the development of neurological diseases^33,34^, Asph is associated with the proliferation, invasion, and metastasis of breast tumor cells^35^, and IL-27 is produced by macrophages, and its receptor IL-27Ra was observed on the surface of CD8^+^ T cells^36^. IL-27 is a member of the IL-12 family of heterodimeric cytokines consisting of the Epstein-Barr virus-induced gene 3 and *p28* chains. The latter of these factors is the limiting factor for producing biological IL-27^37^. Specifically, five AU-rich element ATTTA pentamer sequences were identified in the murine transcript, and four were found in the human transcript of IL-27 *p28*, but not EBI3 (Supplementary Tables S3 and S4). Notably, *p28* was downregulated in the Old WT and Old G3 Terc^*−/−*^ groups compared to the Young WT group (Fig. 6b). Consistently, IL-27 level in the serum of senescent mice after kidney transplantation was lower than that in the Young WT group, while levels of IL-10, IL-12 and IL-23 were low in serum and comparable between these groups (Fig. 6c). This further suggested that IL-27 may be the downstream molecule of Zfp36.

To confirm the regulation of Zfp36 on IL-27 *p28* mRNA, we conducted validation of BMDMs from young and senescent mice in vitro. *p28* mRNA was significantly reduced in senescent mouse BMDMs, both at rest and upon stimulation by Lipopolysaccharide (LPS) (Fig. 6d). To determine if Zfp36 impacted on *p28* mRNA stability, we measured *p28* mRNA degradation after blocking de novo RNA synthesis using actinomycin D (ActD). The degradation of *p28* mRNA was enhanced in senescent mice BMDM, while *beta-Actin (ACTB)* lacking ARE had no significant difference (Fig. 6e), indicating posttranscriptional regulation of *p28*. The interaction between Zfp36 and *IL-27* mRNA was also validated by photoactivatable ribonucleoside‐enhanced individual nucleotide resolution crosslinking and immunoprecipitation (PAR‐iCLIP) in previous study^38^ (Fig. 6f). Consistent with this, immunofluorescence of Zfp36 co-stained with IL-27 *p28* fluorescence in situ hybridization (FISH) also revealed the colocalization of these two molecules (Fig. 6g), which was consistent with a previous study confirmed by RNA Binding Protein Immunoprecipitation (RIP)^39^. Not surprisingly, the levels of IL-27 in the supernatant of senescent mouse BMDMs and irradiated BMDMs were reduced (Fig. 6h, i). In addition, Lyz2-cre;Zfp36f/f mice exhibited a higher-level IL-27 compared to WT, rather than IL-12 and IL-23 (Fig. 6j). MK2 phosphorylation of Zfp36 S52 and S178 residues creates a functional 14-3-3 binding site to avoid binding to target mRNAs, resulting in less degradation of these mRNAs. Therefore, we utilized MK2 inhibitor PF 3644022 and MK2 inhibitor III to enhance the function of Zfp36 in young macrophages^40^. Treatment with PF 3644022 or MK2 inhibitor III resulted in decreased IL-27 *p28* expression level and reduced IL-27 in the supernatant of BMDMs (Supplementary Figs. S8a, b and S9a, b), due to accelerated degradation (Supplementary Figs. S8c and S9c), while Tomivosertib, an irrelevant kinase inhibitor, showed comparable level as dimethylsulfoxide (DMSO). We next cultured naive CD8^+^ T cells with the supernatant from BMDMs. The supernatant from BMDMs treated with PF 3644022 or MK2 inhibitor III resulted in attenuation of IFN-γ^+^CD8^+^ T cells compared to BMDMs treated with DMSO (Supplementary Figs. S8d and S9d). CD8^+^ T cell proliferation was also lowered upon culturing with supernatants of BMDMs treated with PF 3644022 or MK2 inhibitor III (Supplementary Figs. S8e and S9e). To further confirm if Zfp36 knockdown in senescent macrophages could restore IL-27 production and promotion of CD8^+^ T cell activation and proliferation, we silenced Zfp36 expression using siRNA in BMDMs from Old G3 Terc^*−/−*^ mice (Supplementary Fig. S10a). As expected, the mRNA of IL-27 *p28* was restored upon silencing of Zfp36 (Supplementary Fig. S10b) due to reduced degradation (Supplementary Fig. S10c). The level of IL-27 was also higher in its supernatant (Supplementary Fig. S10d). Supernatants from BMDMs silencing of Zfp36 promoted CD8^+^ T cell activation and proliferation, which was suppressed upon IL-27 blockade with a neutralizing antibody (Supplementary Fig. S10e, f). These data demonstrated that overexpression of Zfp36 in senescent mouse macrophages accelerated IL-27 *p28* mRNA degradation and reduced IL-27 release, which led to CD8^+^ T cellular dysfunction.

### Disrupted IL-27 signaling on CD8^+^ T cells or neutralizing antibody to IL-27 in conditional macrophage Zfp36 KO mice reverses acute renal allograft rejection

**Fig. 7:**
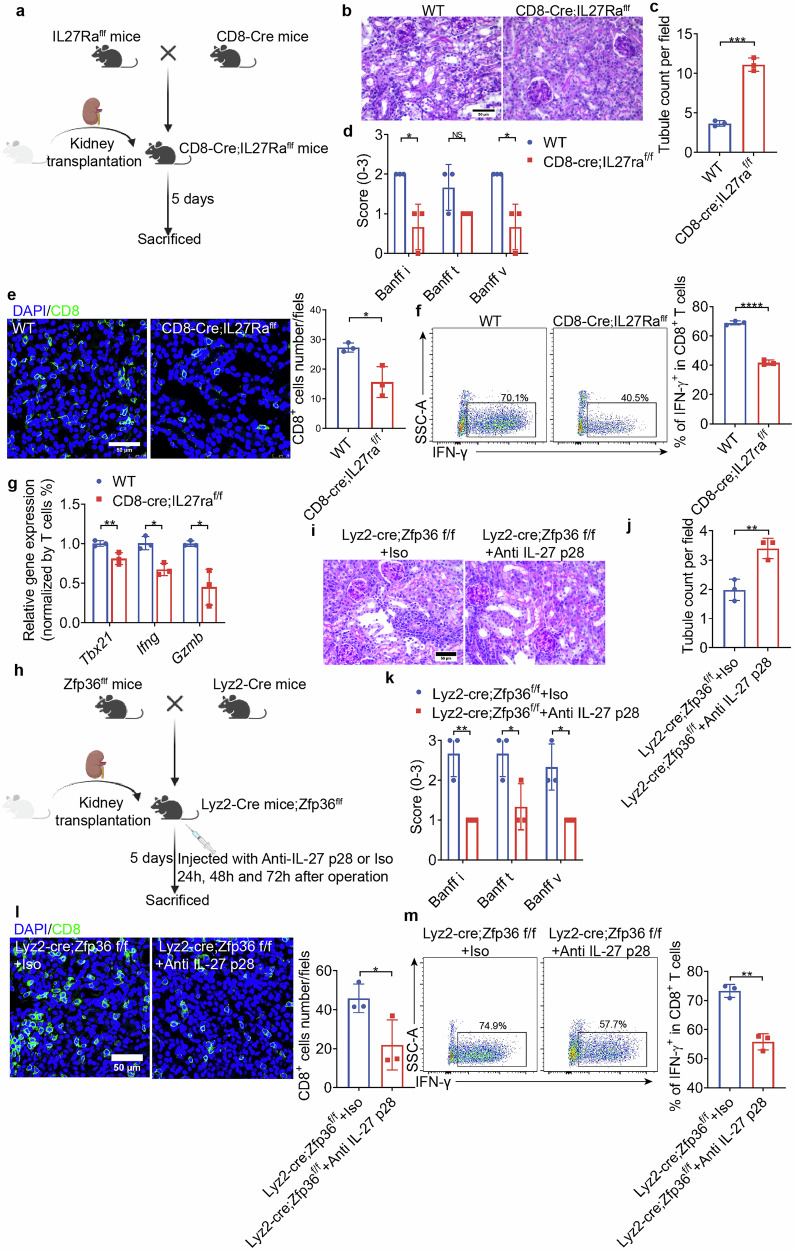
CD8-cre;IL27Ra^f/f^ mice exhibited alleviated acute rejection, while neutralization of IL-27 attenuated aggravated acute rejection in Lyz2-cre;Zfp36^f/f^ mice. **a** Schematic diagram of CD8-cre;IL27Ra^f/f^ mouse experimental design. **b** Representative image of PAS staining of allografts. Scale bar, 50 μm. **c** Quantitative analysis of healthy tubule count per field, *n* = 3/group. **d** Quantitative analysis of i, t and v scores according to Banff 2019, *n* = 3/group. **e** Representative image of CD8^+^ T cells (CD8, green) immunofluorescent staining of allografts. Scale bar, 50 μm. Quantitative analysis of CD8^+^ T cells number per field is shown on the right side, *n* = 3/group. **f** Representative flow panels of IFN-γ^+^CD8^+^ T cells from allografts are shown. Quantitative analysis of the IFN-γ^+^CD8^+^ T ratios is shown on the right side, *n* = 3/group. **g** Expression level of *Tbx21*, *Ifng*, *Gzmb*, and *Prf1* normalized for the numbers of T cells in allografts analyzed using qPCR, *n* = 3/group. **h** Schematic illustration of anti-IL-27 p28-treated Lyz2-cre;Zfp36f/f mouse experimental design. **i** Representative image of PAS staining of allografts. Scale bar, 50 μm. **j** Quantitative analysis of healthy tubule count per field, *n* = 3/group. **k** Quantitative analysis of i, t and v scores according to Banff 2019, *n* = 3/group. **l** Representative image of CD8^+^ T cells (CD8, green) immunofluorescent staining of allografts. Scale bar, 50 μm. Quantitative analysis of CD8^+^ T cells number per field is shown on the right side, *n* = 3/group. **m** Representative flow panels of IFN-γ^+^CD8^+^ T cells from allografts are shown. Quantitative analysis of the IFN-γ^+^CD8^+^ T ratios is shown on the right side, *n* = 3/group. Data are presented as mean ± SD. Statistical analysis was performed using two-tailed Student’s *t*-test (**b**–**h**, **j**–**l**). *P < 0.05, **P < 0.01, ***P < 0.001, ****P < 0.0001.

To further explore the necessary role of IL-27 signaling on CD8^+^ T cell immunity during renal allograft rejection, we established a TCMR model by conditionally deleting IL-27Ra from CD8^+^ T cells (Fig. 7a). It caused no other CD8^+^ T cell defects in number, migration, homeostasis or maturation (Supplementary Fig. S11). PAS staining revealed that the graft in Cd8-cre;IL27Raf/f mice remained more intact renal tubules and showed lower Banff i, v scores 5 days after surgery (Fig. 7b–d). CD8^+^ T cell infiltration was reduced in Cd8-cre;IL27Raf/f mice compared to WT mice (Fig. 7e), and the IFN-γ^+^CD8^+^ T cell ratio was also reduced (Fig. 7f). Consistently, the expression of *Tbx21*, *Ifng*, *Gzmb*, and *Prf1* was significantly decreased in the allograft of knockout mice (Fig. 7g). To confirm the role for senescent macrophages in renal allograft rejection by inhibiting via IL-27 signaling on CD8^+^ T cells, we blocked IL-27 with a neutralizing antibody or adding IL-27 to BMDMs supernatant. Addition of IL-27 neutralizing antibody to the supernatant from young BMDMs resulted in strong repression of CD8^+^ T cell activation and proliferation, which was restored by adding IL-27 to senescent BMDMs (Supplementary Figs. S12 and S13). This was further validated in vivo in conditional macrophage Zfp36 KO mice in which blockade of IL-27 with a neutralizing antibody (Fig. 7h) reversed the severity of renal allograft rejection by significantly increasing the residual healthy tubules, lower Banff i, t, v scores (Fig. 7i–k) and inhibiting excessive CD8^+^ T cell infiltration (Fig. 7l) and IFN-γ^+^CD8^+^ T ratio (Fig. 7m).

### Serum IL-27 levels were elevated and positively correlated with telomere length in patients with acute cellular rejection of kidney transplantation

**Fig. 8:**
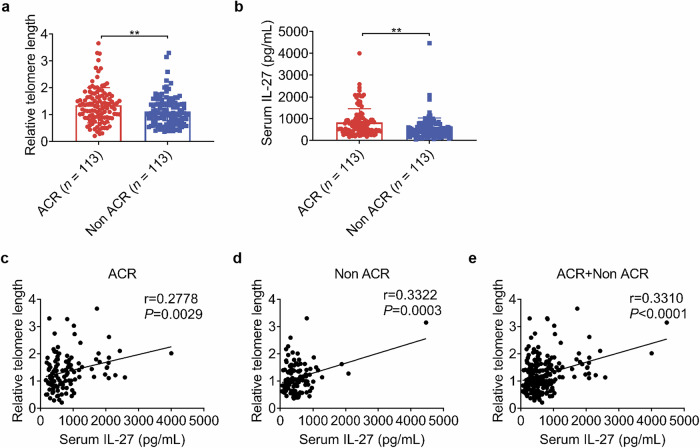
Serum IL-27 was elevated and positively correlated with telomere length in patients with ACR of kidney transplantation. **a** Relative telomere length of PBMCs collected at the time of diagnosis with ACR or routine follow-up in ACR and Non-ACR patients using T/S methods, *n* = 113/group. **b** Serum IL-27 levels in ACR and Non-ACR patients measured by ELISA. **c** Pearson correlation analysis between relative telomere length of PBMCs and serum IL-27 level in ACR patients. **d** Pearson correlation analysis between relative telomere length of PBMCs and serum IL-27 level in Non-ACR patients. **e** Pearson correlation analysis between relative telomere length of PBMCs and serum IL-27 level in all patients. Data are presented as mean ± SD. Statistical analysis was performed using two-tailed Student’s *t*-test (**a**, **b**) and Pearson correlation analysis (**c**–**e**). **P < 0.01. ACR, acute cellular rejection. Non-ACR, non-acute cellular rejection.

Finally, we examined the clinical relevance of serum IL-27 in kidney transplantation patients. We measured the relative telomere length of PBMCs collected from patients diagnosed with ACR at the time of rejection and compared it with stable recipients during follow-up. We found that the relative telomere length of PBMCs in the ACR group was longer than that in stable recipients (Fig. 8a), indicating potential differences in immune cell aging between these groups at the point of rejection. Additionally, serum IL-27 levels were elevated in the ACR group compared to stable recipients (Fig. 8b). Furthermore, we found a positive correlation between serum IL-27 levels and relative telomere length in all patients, regardless of status (Fig. 8c–e). These data further support our findings that IL-27 is downregulated in senescent recipients and is associated with a lower occurrence of rejection.

## Discussion

Although the relationship between recipient senescence and graft prognosis has been discussed in previous studies^5–7^, the exact mechanisms of age-dependent alterations impacting rejection and long-term graft survival remain poorly understood. To address this gap in knowledge, we conducted a comprehensive investigation using a single-cell view of acute TCMR in senescent mouse kidney allografts. Our analysis revealed that T cells were the predominant immune cells in the allografts of senescent recipients, with macrophages as the second most abundant cell type, consistent with previous findings in young recipients^23,41^. Interestingly, we observed that CD8^+^ T cell dysfunction was a key factor in the attenuated rejection in senescent recipients, and this dysfunction was regulated by senescent macrophages. Previous studies have suggested the existence of an immunosuppressive network in senescent individuals, with regulatory cells such as myeloid-derived suppressor cells (MDSCs) and M2 macrophages playing a role in suppressing the cytotoxic function of CD8^+^ T cells and impairing their immune surveillance function^42^. The immune function of CD8^+^ T cells was also modulated by macrophages in both tumor and transplantation models^43,44^.

Senescence-associated genes, such as the CDK2 inhibitor p21WAF1/Cip1 (*CDKN1a*) and the CDK4/6 inhibitor p16INK4A (*CDKN2a*) accumulate in senescent cells^25^. Overexpression of p21 has been linked to cellular senescence manifestations, including increased cell volume, elevated SA-β-gal activity, decreased Lamin B1 expression, reduced cell proliferation, and senescence-associated secretory phenotype (SASP)^45^. Studies in obesity models have shown that p21^high^ preadipocytes and macrophages accumulate in the adipose tissue of obese mice and contribute to insulin resistance^46^. In our study, we also observed the accumulation of p21^high^ macrophages in the allografts of senescent recipients, and these senescence macrophages highly expressed Zfp36. Interestingly, a co-expression network analysis of senescence genes (SnGs) in 50 human tissues revealed that *Cdkn1a* (the gene encoding p21) and *Zfp36* were the top two conserved nodes with the widest distribution across tissue modules. This further supports the involvement of macrophages, rather than T cells, as a key cell type associated with the SnGs in the overall network^47^. Consistent with these findings, our data indicated that the alteration of Zfp36 in senescent macrophages led to the dysfunction of CD8^+^ T cells and subsequently resulted in attenuated rejection.

Zfp36 is a well-known RNA binding protein (RBP) that plays a crucial role in destabilizing mRNA targets containing specific sequence motifs known as AREs. These AREs are commonly found within the 3′UTR of many inflammatory mRNAs^48^. Studies in Zpf36-deficient mice have shown various manifestations, including marked medullary and extramedullary myeloid hyperplasia associated with cachexia, erosive arthritis, dermatitis, conjunctivitis, glomerular mesangial thickening, and high levels of anti-DNA and antinuclear antibodies^29^. Additionally, myeloid-specific Zfp36 deficiency in mice resulted in extreme sensitivity to LPS^49^. Despite these well-documented effects of Zfp36 in other contexts, its role in kidney transplantation has remained unknown. Our study has shed light on this aspect, revealing that overexpression of Zfp36 in senescent macrophages plays a critical role in attenuating CD8^+^ T cell activation and proliferation by regulating the stabilization of IL-27.

IL-27 is a cytokine with diverse immunomodulatory functions, including enhancing the function of Th1 and CD8^+^ T cells while inhibiting the activation of Th2, Th17, and Treg cells^36,50^. In our study, we found that the moderated rejection observed in senescent recipients was not dependent on Th1, Th2, Th17, or Treg cells, as their levels were similar. Instead, the stimulatory effect of IL-27 was most prominent in CD8^+^ T cells, where it enhanced proliferation and expression of T-bet, EOMES, and IL-12Rβ2, leading to increased production of IFN-γ and cytolytic activity^51–53^. IL-27 was further confirmed to promote tumor-specific cytotoxic T cell responses and tumor regression in vivo^54,55^. In hematopoietic stem cell transplantation, elevated serum IL-27 levels were associated with transplant failure^56^, while blockade of IL-27 signaling attenuated graft-versus-host disease (GVHD) in mice^57^. In contrast, another study showed mice with IL-27 p28 deficiency in DC exhibited impaired Treg cell function and enhanced effector T cell responses, related to aggravated GVHD in mice^58^. For solid organ transplantation, IL-27 and IL-27R have been reported as key factors for distinguishing rejection individuals in skin and kidney transplantation^59,60^. IL-27Rα^+^ cells promoted skin allograft rejection through enhancing alloreactive proliferation, inhibiting apoptosis and up-regulating IFN-γ via enhancing STAT pathway^61^. In line with these research, CD8-cre;IL27Raf/f mice showed alleviated rejection, and serum IL-27 levels were elevated and positively correlated with telomere length in patients with acute cellular rejection. Neutralization of IL-27 attenuation of aggravated acute rejection in Lyz2-cre;Zfp36f/f mice further confirmed the macrophage Zfp36/IL-27 axis regulating CD8^+^ T cells in senescent recipients. However, another study showed that IL-27 plays a role in tolerance by inducing IL-10-expressing CD4^+^ T cells in rat cardiac allograft^62^. This inconsistency may be explained by differences in observation time point and transplanted organs.

Other cytokines of IL-12 family were also evaluated. Zfp36 was predicted to bind to IL12 and reported to inhibit IL23 production^30,31^. However, our data showed that IL-12 and IL-23 levels were low in TCMR and thus we suppose that they may not have a sufficient impact on rejection.

**Fig. 9:**
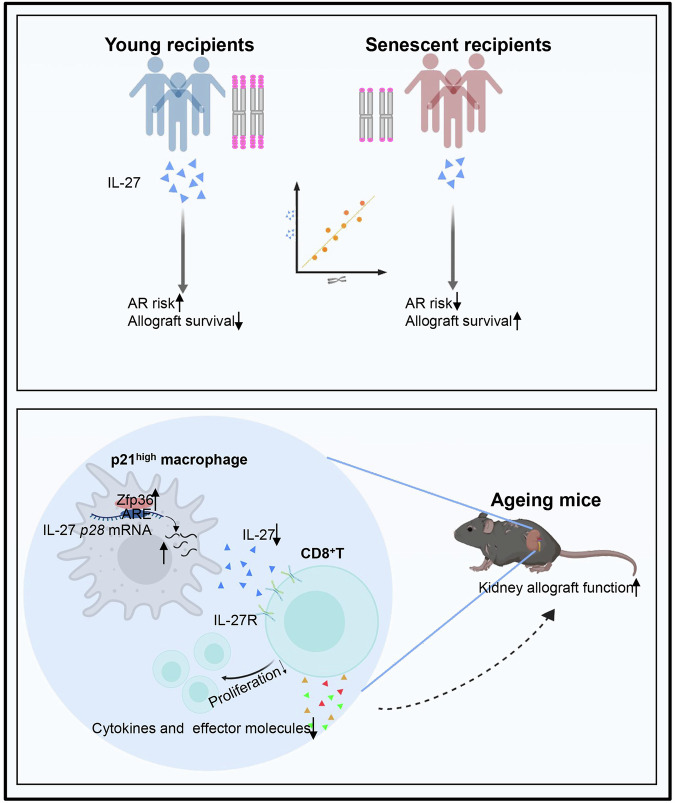
Overall summary of the study. Senescent recipients developed less graft rejection after kidney transplantation. Increased expression of Zfp36 in senescent p21^high^ macrophages resulted in reduced IL-27 production, ultimately leading to CD8^+^ T cell dysfunction and attenuated rejection.

Our study has provided insights into a regulatory age-dependent pathway involving the macrophage Zfp36/IL-27 axis and its impact on CD8^+^ T cells in senescent recipients. We demonstrated that increased expression of Zfp36 in senescent p21^high^ macrophages resulted in reduced IL-27 production, ultimately leading to CD8^+^ T cell dysfunction and attenuated rejection (Fig. 9). These findings highlight the potential for more personalized immunosuppressive approaches for elderly transplant recipients to avoid inappropriate immunosuppression. This study has also provided an invaluable tool related to the macrophage Zfp36/IL-27 axis and IL-27Ra on CD8^+^ T cells for future precision medicine approaches.

Indeed, our study does have specific limitations that warrant consideration. One major limitation is that scRNA-seq was performed on mouse models, mainly due to the scarcity of available kidney biopsy samples from human patients and the challenges in obtaining sufficient quantities of high-quality cells from these samples. Although the protein sequence conservation between humans and mice is relatively high, at around 85%^63^, future sequencing of human patient samples would provide more valuable and directly relevant information to human kidney transplantation. In addition, IL-27 may enhance or suppress NK function depending on the context^64^. It is also reported that immune-tolerant DCs are generated by IL-27^65^. The current data do not rule out the potential side effects of anti-IL-27 on other immune cells after kidney transplantation, which is valuable for therapeutic potential of IL-27 antibody and worth further exploration. Another limitation is the lack of clarity on how senescence induces Zfp36 overexpression in macrophages. Understanding the precise mechanisms underlying this phenomenon would be crucial in fully unraveling the regulatory pathway involved in the observed alterations in CD8^+^ T cell function and transplantation outcomes. Given the complexity and diversity of alterations in the immune system linked to senescence, further studies are required to understand the process underlying kidney transplantation rejection completely and precisely in senescent recipients.

## Materials and Methods

### Patient specimens

Studies involving human participants received approval from the Research Ethics Committee of the First Affiliated Hospital, Zhejiang University School of Medicine (2024-307) and Hannover Medical School (the Ethical board No. 2765). Written informed consent was obtained from the patients/participants (or their next of kin) who participated in this research. All clinical and research activities being reported are consistent with the Principles of the Declaration of Istanbul as outlined in the “Declaration of Istanbul on Organ Trafficking and Transplant Tourism” and complied with the Declarations of Helsinki Medical on Research Involving Human Subjects. We included a total of 119 patients diagnosed with acute T cell-mediated rejection using Banff guidelines, alongside 119 gender and operation time matched patients without any rejection in ZJU, China cohorts. Study flow chart was provided in Supplementary Fig. S1a. Demographics and baseline characteristics of recipients and donors, donor–recipient matching information and immunosuppression therapy were shown on Supplementary Table S1. Banff type of rejection was provided on Supplementary Table S2. Before and at the time of TCMR diagnosis, PBMC and serum samples were collected from the participants. For graft survival analysis, graft loss was defined as need for dialysis or retransplantation, or death with a functioning graft.

### Animal model

Mice around 18 months old are considered as ageing mice^66^. Terc^*−/−*^ mice were provided by Zhenyu Ju (Jinan University, China) and were bred to the third generation. Absence of Terc (RNA component of telomerase) leads to telomere shortening progressively during successive generations of Terc deficient mice^19^. G3 Terc^*−/−*^ mice develop the senescence phenotype earlier than WT mice after birth. The life span of G3 Terc^*−/−*^ mice is about 1.5 years. Even if they lived to 18 months, they were too weak to survive from surgery. Thus, 12 months G3 Terc-/- mice was employed in this study. IL27Raf/f mice were mated with Cd8-cre mice, purchased from Cyagen (Jiangsu, China). Lyz2-cre;Zfp36f/f mice were purchased from Cyagen. Ectopic renal transplantation was conducted by transplanting kidneys from male BALB/c mice (H‐2 d; 8 weeks old) into male C57Bl6/J background recipients as previously described^67^. Mice were euthanized 5 or 7 days after the surgery. For macrophage depletion, 10 μL/g Clodronate Liposome (Liposome, the Netherlands) was administered intraperitoneally 48 h after surgery. For neutrophil depletion, 400 μg InVivoMAb anti-mouse Ly6G (BioXCell, USA) was administered intraperitoneally 48 h after surgery. For IL-27 p28 neutralization, 40 μg of anti-IL-27 p28 (Biolegend, USA) was administered via the orbital vein 24 h, 48 h, and 72 h after surgery. All mice were housed in the animal center of Zhejiang University according to animal care regulations. As mentioned above, the Research Ethics Committee of the First Affiliated Hospital, College of Medicine, Zhejiang University, approved the experimental protocols.

### Cellular Assays

HK2 cells were obtained from the American Type Culture Collection. BMDMs were generated from Young WT, Old WT and Old G3 Terc^*−/−*^ mouse bone marrow cells and cultured in a complete RPMI1640 medium containing 10% (v/v) FBS, 1% (v/v) Penicillin/Streptomycin, and 10 ng/mL of M-CSF (Novoprotein, China) for 1 week.

### Telomere length measurements

PBMC DNA was isolated from peripheral blood leukocytes using the AxyPreP Blood Genomic DNA miniprep Kit (Axygen, Hangzhou, China) according to the manufacturer’s instructions. Telomere length measurements of the DNA were conducted using the telomere to single copy gene (T/S) ratio protocol^68^. The gene-specific PCR primers are indicated in Supplementary Table S5.

### PAS staining, residual renal function assessment and Banff score

Kidney tissues were fixed in 4% formaldehyde buffer before being dehydrated, embedded in paraffin, sectioned (2 µm sections), and stained using PAS for histological analysis. Images were obtained using a 40× objective lens by immunofluorescence microscopy (Lecia, DM4000, Germany). Healthy tubules were defined by the presence of an intact basement membrane, intact tubule lumen, healthy cytoplasmic volume, and a maintained apical microvilli brush border, all of which reflected functioning nephron mass and were quantified by counting the average number of tubules per field^69^. According to the Banff 2019 TCMR diagnostic criteria^21^, renal interstitial inflammation (i), tubulitis (t), and intrarenal arteritis (v) were scored.

### Immunofluorescence (IF) staining and confocal microscopy

The kidney tissues were fixed in 4% paraformaldehyde at 4 °C for 24 h. After dehydration in 30% sucrose overnight at 4 °C, the kidneys were embedded in OCT and sectioned to generate 8 μm-thick sections. Sections were permeabilized using 0.2% TritonX-100 for 5 min and blocked with 5% BSA for 1 h at room temperature. Cells were then incubated with a primary antibody against Cd8a (1:200 dilution in 5% BSA), F4/80 (1:200 dilution in 5% BSA), and Zfp36 (1:100 dilution in 5% BSA). On the second day, after three washes with PBS, sections were incubated with a 1:500 dilution in 5% BSA of Alexa-488 or -594 conjugated secondary antibody. The stained sections were provided with a final wash and mounted with Prolong Gold antifade reagent and DAPI (Abcam). Images were obtained using a 20× objective lens by confocal microscopy (Nikon A1, Nikon, Japan). For CD8^+^ T cell infiltration, the CD8^+^ cells were counted in each field. For semiquantitative analysis of the Zfp36 expression level, fluorescence intensity was normalized to F4/80.

### Flow cytometry

The kidneys were digested and filtered using a 70-μm cell strainer to generate single-cell suspensions. After enrichment, the immune cells were stimulated using a cell stimulation cocktail with protein transport inhibitors (eBioscience, USA) for 4 h. Fixation and permeabilization were conducted for intracellular and endonuclear staining. Cells were stained with the following antibodies: CD3-Pe-Cy7 (Invitrogen, USA), CD8-FITC (Biolegend), Foxp3-APC (Invitrogen), IFN-γ-APC (Invitrogen), IL4-PE (Invitrogen), IL17-PE (Invitrogen), p21-FITC (Abcam), F4/80-Pe-Cy7 (Biolegend), and Cd11b (Invitrogen). Finally, flow cytometric data acquisition was performed using a BD CantoII (BD, USA) and analyzed with FlowJo (BD) software.

### RNA extraction and qPCR

RNA was isolated using TRIzol® reagent (Invitrogen, USA). PrimeScript™ II Reverse Transcriptase (Takara, Japan) was utilized to reverse transcribe 1000 ng of RNA to create the cDNA for each sample. Before qPCR, we diluted the first-strand cDNA synthesis products at 1:4 for each sample. A two-step PCR reaction was performed as follows: pre-denaturation at 95 °C for 30 s; PCR reaction consisting of 40 cycles of denaturation at 95 °C for 5 s, annealing at 60 °C for 30 s; followed by dissociation at 95 °C for 15 s, 60 °C for 1 min, and 95 °C for 15 s. The relative mRNA expression of the target genes was normalized to *GAPDH* from the same sample. Results were expressed as the fold change in expression, and values were calculated as the ratio of target gene expression to control gene expression. Gene-specific primers were designed using Primer-Blast (Supplementary Table S6). The efficiency of the primers was estimated through Ct < 30 and the presence of no multiple Tm peaks. Real time-PCR reactions were conducted using SYBR Green reagent and a Real-Time PCR Detection System (Life, USA).

### Droplet-based scRNA-seq and analysis of scRNA-seq data

Single-cell suspensions were prepared using a Multi Tissue Dissociation Kit2 (MiltenyiBiotec, Germany) following the manufacturer’s protocol. The single-cell samples (5000 to 10,000 cells/sample) were subjected to a 10× Chromium Single Cell Instrument (10× Genomics) for barcoding and cDNA library construction. Sequencing was conducted by CapitalBio Technology Co., Ltd (Beijing, China).

We converted 10× Genomics-generated reads to fastq, aligned reads to the mm10 reference genome, filtered, and counted using the 10× Genomics’ CellRanger pipeline. Subsequent steps were performed using R software (Version 4.1.2). After constructing Seurat objects for each sample, we excluded cells expressing fewer than 200 or greater than 7500 genes, those with a mitochondrial UMI rate higher than 15%, and those with a UMI count lower than 500 or greater than 10,000. Normalization and batch effect removal were then conducted. We acquired the unsupervised cell cluster based on the top 25 Principal Component Analysis (PCAs) and used *t*-SNE for dimensional reduction and clustering. We separated the cells into eight clusters based on known markers, including T cell, macrophage, renal parenchymal cell, granulocyte, NK, B cell, NKT, and DC. To identify differentially expressed genes (DEGs) between groups, the Seurat package FindMarkers using the Wilcoxon rank sum test algorithm was employed with the following criteria: 1) |logfc.threshold | > 0.25; 2) *P* value < 0.05; 3) min.pct > 0.1.

CellChat was applied to assess the major signaling inputs and outputs among all cell clusters^70^. Specifically, CellChat objects were constructed independently for each sample and merged. The number of interactions between different cell clusters was visualized using the netVisual_diffInteraction function.

To evaluate the cytotoxic function, we used tidyverse package to determine the cytotoxic score based on the expression of *Prf1*, *Ifng*, *Nkg7*, *Gzma*, *Gzmb*, *Cst7*, and *Tnfrsf10*^24^.

The data after SCTransform were used for expression cutoff analysis. For all cell types, “exprs$Cdkn1a > 0” is defined as p21^high^ and “exprs$Cdkn1a < 0” is defined as p21^low^. For macrophages, “exprs$Zfp36 > 0” is defined as Zfp36high and “exprs$Zfp36 < 0” is defined as Zfp36^low^.

### CD8^+^ T cell Isolation and culture

Naive CD8^+^ T cells were isolated from the spleen using the mouse naive CD8^+^ T cell isolation kit (STEMCELL Technologies) following the manufacturer’s instructions. Sorted cells were resuspended and seeded into 24-well plates coated with 3 μg/mL anti-CD3 (Biolegend) and co-cultured with supernatants collected from activated macrophages in the presence of 6 μg/mL CD28 (Biolegend). For neutralization testing or supplementation testing, 5 ng/mL of anti-IL-27 p28 (Biolegend) and 5 μg/mL of murine recombinant IL-27 was added to supernatants. After 72 h, cells were subjected to flow cytometry analysis. An Annexin V-FITC/PI apoptosis kit (Multi Science, China) was utilized for apoptosis testing, following the manufacturer’s protocol. For proliferation testing, sorted cells were labeled using 5 μM CFSE (Invitrogen) and cultured as outlined above. The cells were subjected to flow cytometry analysis after 72 h.

### CD8^+^ T cell migration

Isolated Naive CD8^+^ T cells were seeded to the insert of a 24-well Transwell® plate with 300 ng/mL CCL19 in the lower chamber. The cells migrated into the lower chamber were counted and analyzed by flow cytometry after 12 h of incubation.

### RNA in situ hybridization

The probes and RNA in situ hybridization kit were provided by GenePharma (Shanghai, Chian). The probe sequence was as follows: AAAGTCAGGGAAACATTGGGAAGATGGTATACTTGGATGACACCTGATTGGGGGAGATCCAGCCTCATGGCCCACA. The experiment was conducted according to the manufacturer’s instructions. Thereafter, IF staining occurred, and images were captured on the slides as previously described. To confirm the colocalization of Zfp36 and IL-27 *p28* mRNA, Pearson correlation was calculated by ImageJ (installed with Colocalization Finder plugin). Pearson correlation > 0.3 was considered to be interaction^71,72^.

### Enzyme-linked immunosorbent assay (ELISA)

Cell culture supernatants and serum from transplanted mice and patients were stored at −80 °C. IL-27 was detected using a mouse IL-27 p28/IL-30 Quantikine ELISA Kit (R&D systems, USA) or Human IL-27 DuoSet ELISA (R&D) according to the manufacturer’s instructions. IL-10, IL-12 and IL-23 were detected by Mouse IL-10 Quantikine ELISA Kit (R&D), Mouse IL-12 p70 Quantikine ELISA Kit (R&D) and Mouse IL-23 Quantikine ELISA Kit Summary (R&D), respectively. Concentrations were determined by standard curve regression analysis.

### siZfp36 silencing of Zfp36 in BMDMs

For Zfp36 silencing in BMDMs, siRNA was synthesized by GenePharma. The siRNA sequences for Zfp36 and NC were: siZfp36 sense GAUGCCACUUCAUCCACAATT, siZfp36 anti-sense UUGUGGAUGAAGUGGCAUCTT, siNC sense UUCUCCGAACGUGUCACGUTT, siNC anti-sense ACGUGACACGUUCGGAGAATT. The siRNA was transfected using RNAiMAX (Invitrogen, USA) following the manufacturer’s directions.

### Statistical analysis

Statistical analysis was conducted using Prism software 7 (GraphPad Inc, USA). Results were presented as means ± SD. Statistical analysis was performed using a two-tailed *t*-test, Two-Way ANOVA, and correlation analysis using Pearson’s correlation coefficient. Kaplan-Meier curves tested by a Log-Rank test were used to evaluate the graft survival. *P* < 0.05 was considered to be statistically significant, and the significance was indicated as *P < 0.05, **P < 0.01, ***P < 0.001, ****P < 0.0001.

## Supplementary information

Supplementary Information
