# Supplementary material for: Senescence-induced p21high macrophages contributed to CD8+ T cells-related immune hyporesponsiveness in kidney transplantation via Zfp36/IL-27 axis
Source: Cell Discov. 2025 Apr 15;11:38. doi: 10.1038/s41421-025-00784-2 (PMC12000408; doi:10.1038/s41421-025-00784-2)
Supplement: Supplementary file 1 — Supplementary Information [file 41421_2025_784_MOESM1_ESM.pdf]

Supplementary Information

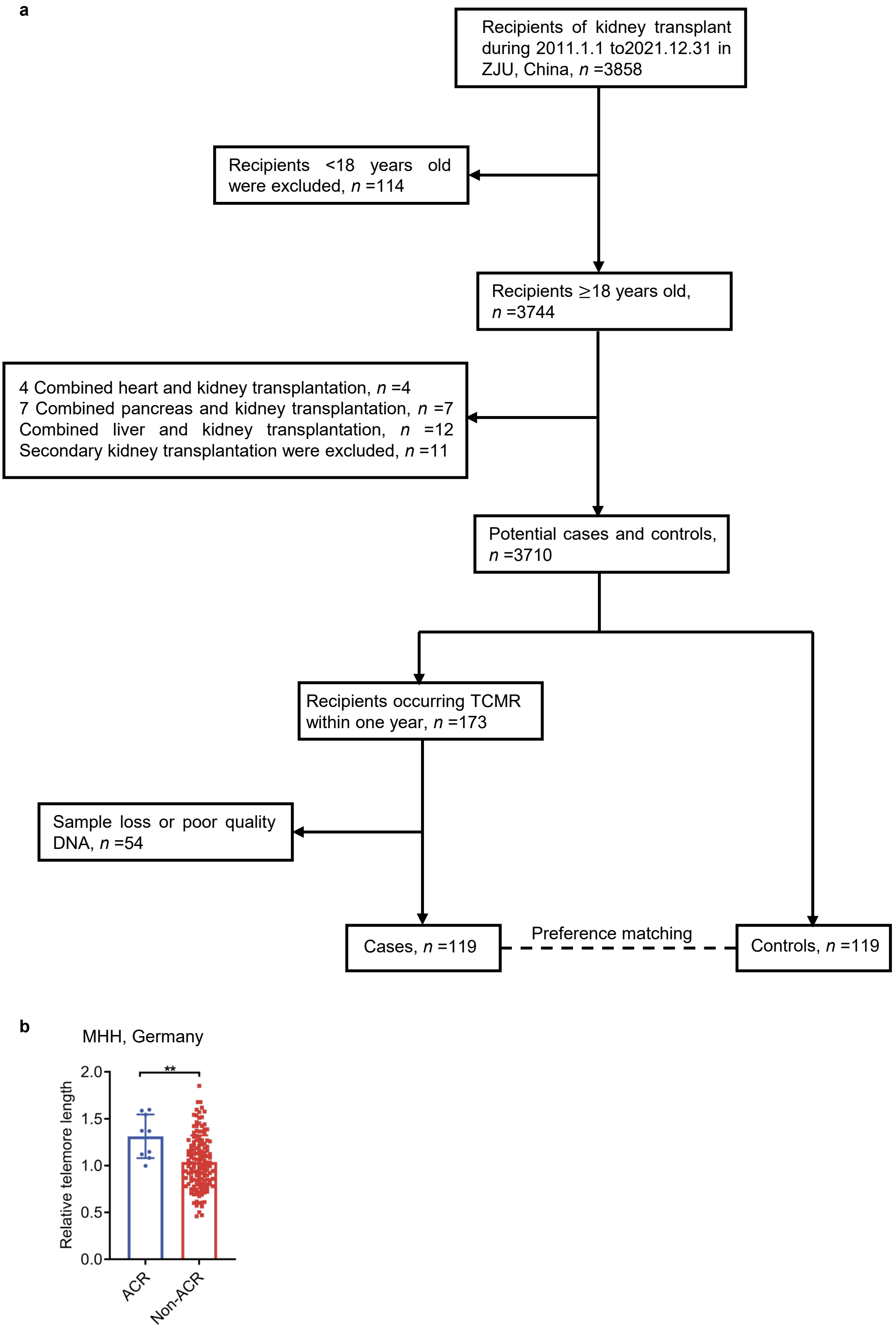

Supplementary Fig. 1 Clinical cohort. a Study flow chart of ZJU, China. **b** Relative telomere length of PBMCs collected before surgery in ACR and Non-ACR patients

from MHH, Germany using T/S methods, $n = 9$ and 143, respectively. Data are presented as mean ± SD. Statistical analysis was performed using Two-tailed Student's T-test (a-l). $^{**}P < 0.01$.

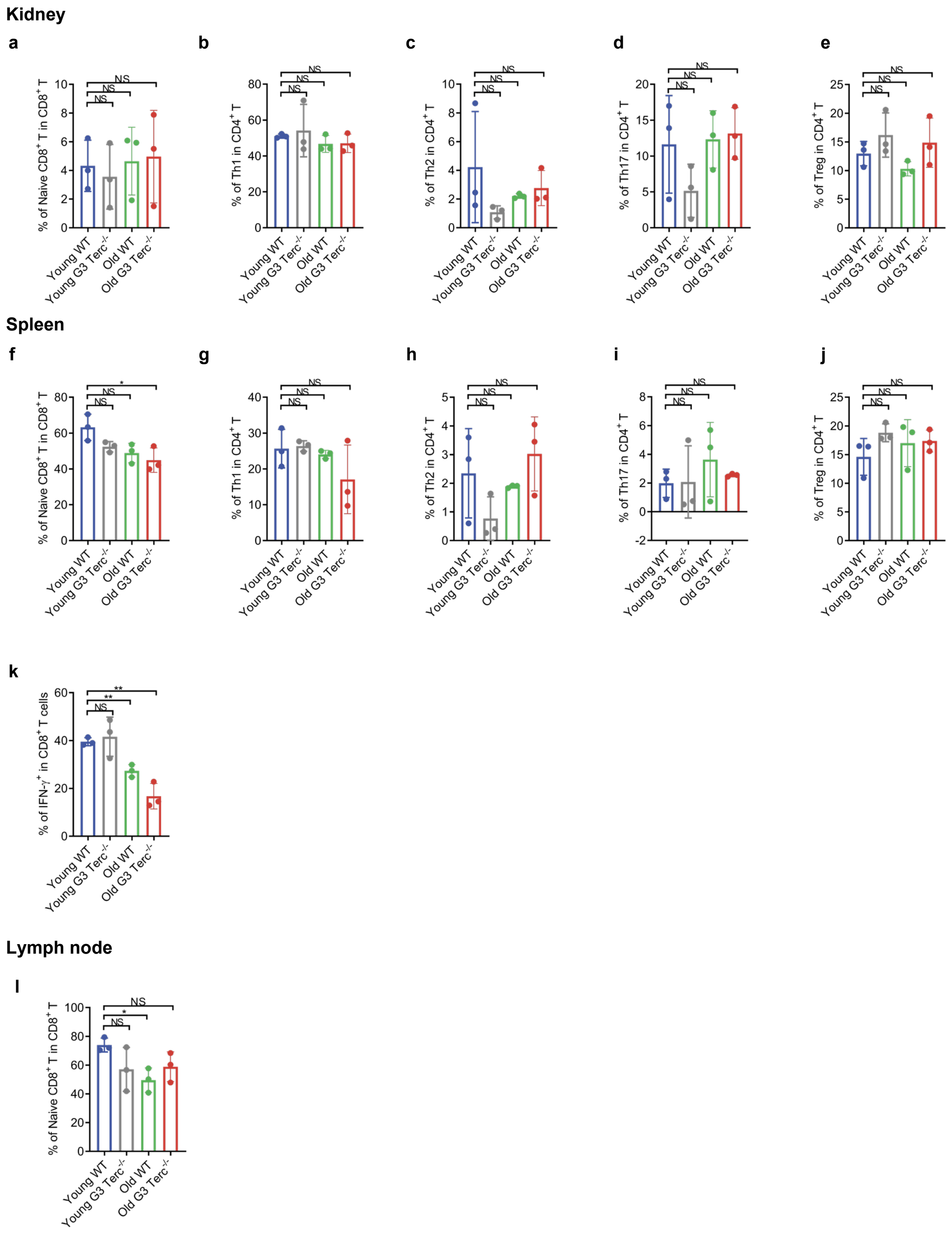

Supplementary Fig. 2 Phenotypes of CD4$^+$ T and CD8$^+$ T cells in kidney, spleen and lymph node. **a** Naïve CD8$^+$ T/CD8$^+$ T ratios in allograft, *n* = 3/group. **b** Th1/CD4$^+$ T ratios in allograft, *n* = 3/group. **c** Th2/CD4$^+$ T ratios in allograft, *n* = 3/group. **d** Th17/CD4$^+$ T ratios in allograft, *n* = 3/group. **e** Treg/CD4$^+$ T ratios in allograft, *n* = 3/group. **f** Naïve CD8$^+$ T/CD8$^+$ T ratios in spleen, *n* = 3/group. **g** Th1/CD4$^+$ T ratios in spleen, *n* = 3/group. **h** Th2/CD4$^+$ T ratios in spleen, *n* = 3/group. **i** Th17/CD4$^+$ T ratios in spleen, *n* = 3/group. **j** Treg/CD4$^+$ T ratios in spleen, *n* = 3/group. **k** IFN-γ^+CD8$^+$ T /CD8$^+$ T ratios in spleen, *n* = 3/group. **l** Naïve CD8$^+$ T/CD8$^+$ T ratios in lymph node, *n* = 3/group. Data are presented as mean ± SD. Statistical analysis was performed using Two-tailed Student's T-test (a-l). *$P < 0.05$, **$P < 0.01$, NS, not significant.

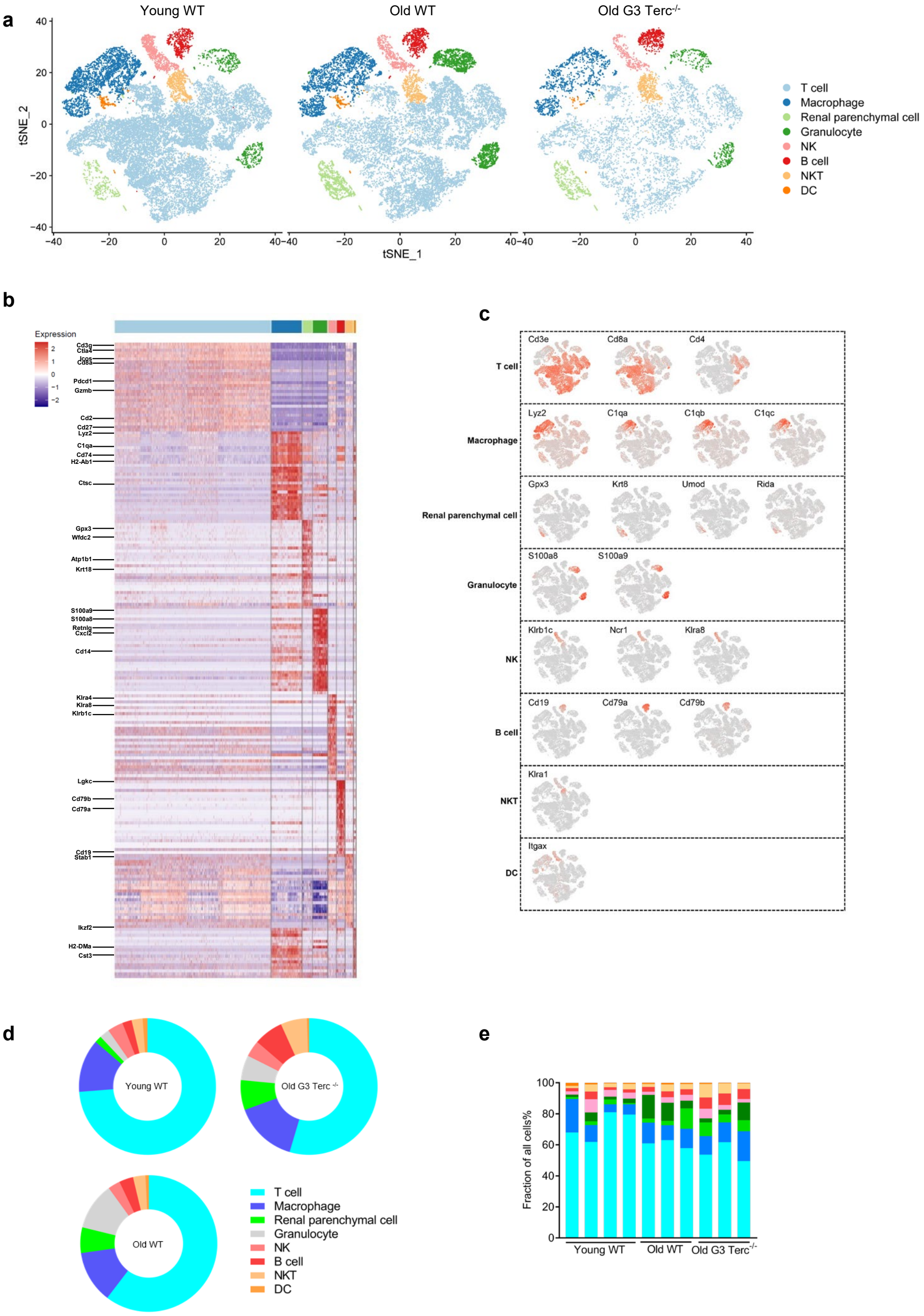

Supplementary Fig. 3 Composition and marker genes across cell types in senescent mice allograft. a t-SNE plot highlighting eight cell clusters divided by group. **b** Heatmap of cell-type-specific marker expression across eight cell clusters. Red denotes high expression, while blue denotes low expression. **c** Marker gene expression

projected on t-SNE plots. Red denotes high expression, while grey denotes low expression. **d** Fraction of cell clusters present in groups. **e** Fraction of cell clusters present in individuals.

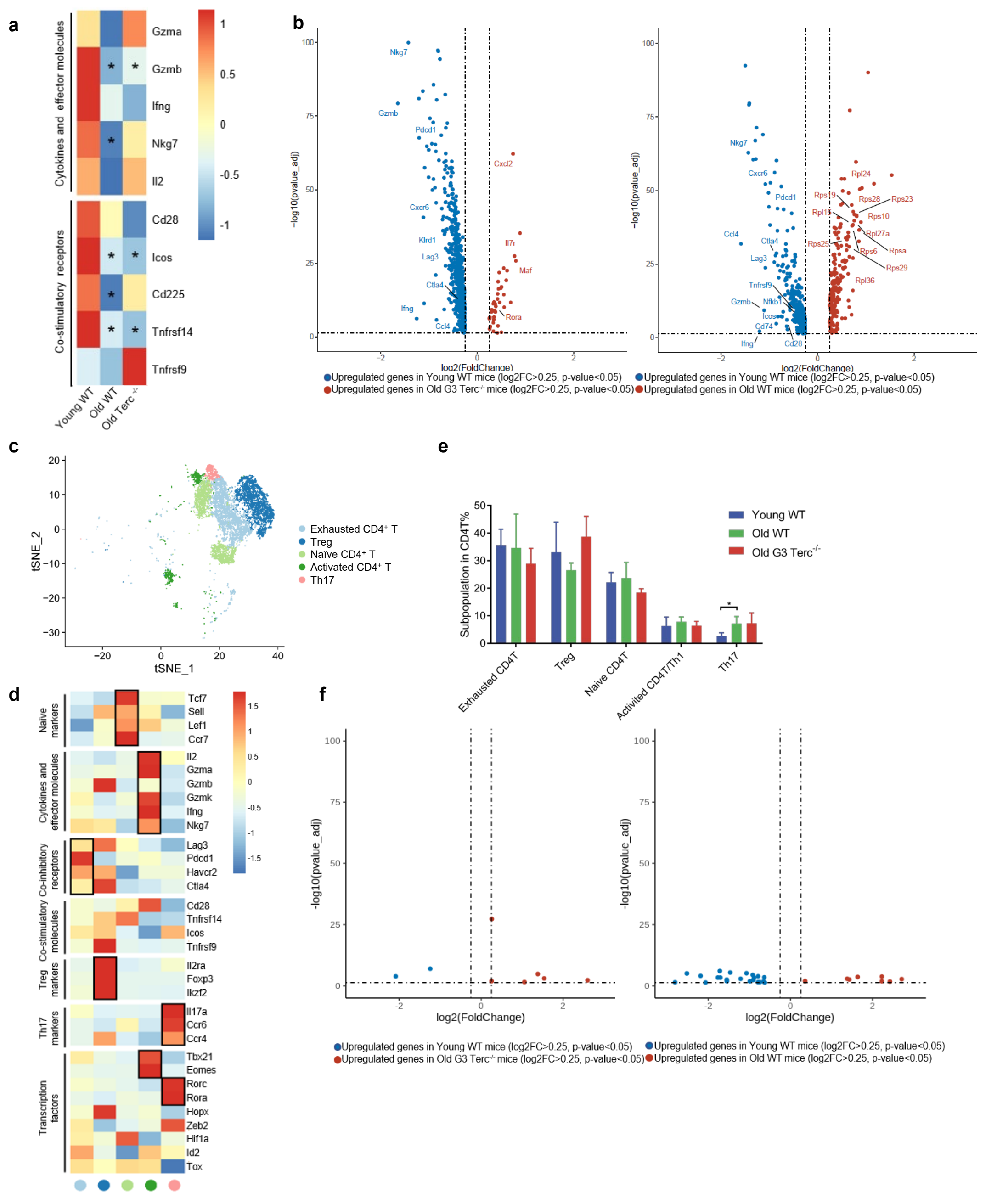

Supplementary Fig. 4 Impaired cytotoxic functioning of Ifng^{high} CD8^{+} T subcluster and minimal change in CD4^{+} T cells in senescent mice. a Normalized mean expression of Ifng high CD8^{+} T subcluster across three groups, asterisks indicate significant differences from Young WT. **b** Volcano plot of the Ifng high CD8^{+} T subcluster illustrating the log2 fold change (x-axis) and -log10 (*P* value_adj) (y-axis) of the differential analysis. Each dot represents a single gene. The dashed horizontal

black line represents a *P* value_adj of 0.05. The dashed vertical black line represents a |log2 fold change| of 0.25. Significant genes (*P* value_adj < 0.05, |log2 fold change|>0.25) are highlighted. The names of the significant genes are displayed alongside the dots. **c** 5 clusters of $CD4^+$ T cells were identified. **d** Normalized mean expression of selected $CD4^+$ T cell function-associated genes within each cell cluster. Black boxes delineate the prominent patterns defining known $CD4^+$ T cell subtypes. **e** Fraction of $CD4^+$ T subclusters across three groups. **f** Volcano plot of the Activated CD4/Th1 subcluster illustrating the log2 fold change (x-axis) and -log10 (*P* value_adj) (y-axis) of the differential analysis. Each dot represents a single gene. The dashed horizontal black line represents a *P* value_adj of 0.05. The dashed vertical black line represents a |log2 fold change| of 0.25. Significant genes (*P* value_adj < 0.05, |log2 fold change|>0.25) are outlined.

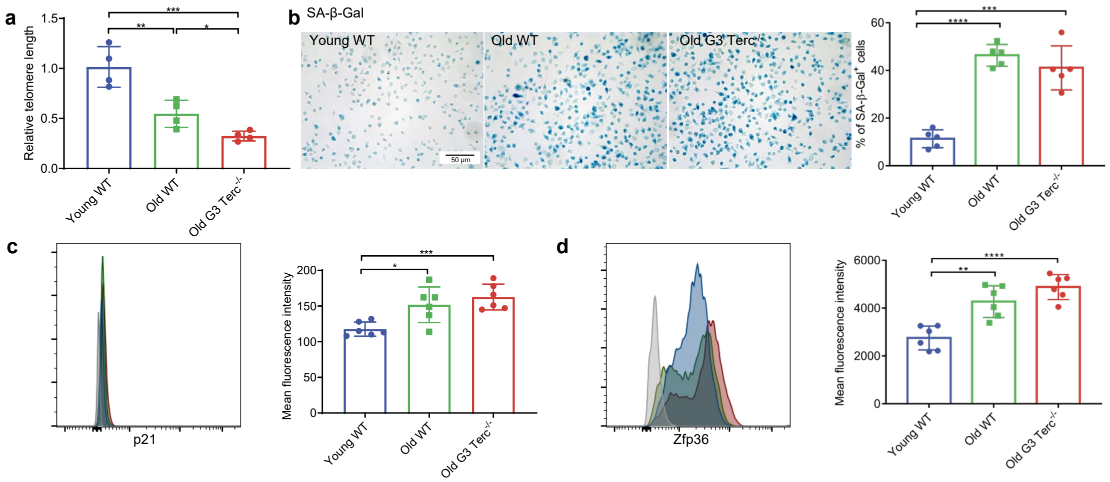

Supplementary Fig. 5 p21 and Zfp36 were upregulated in BMDMs from senescent mice. **a** Relative telomere length of BMDM from Young WT, Old WT, and Old G3 $Terc^{-/-}$ mice, *n* = 4/group. **b** Representative image illustrating SA-β-Gal staining of BMDMs from Young WT, Old WT, and Old G3 $Terc^{-/-}$ mice. Scale bar, 50 μm. Quantitative analysis of SA-β-Gal^+ cells is illustrated on the right side, *n* = 5/group. **c** Representative flow panels of p21 expression in BMDMs. Iso control was shown in grey. Quantitative analysis of mean fluorescence intensity is illustrated on the right side, *n* = 5/group. **d** Representative flow panels of Zfp36 expression in BMDMs. Iso control was shown in grey. Quantitative analysis of mean fluorescence intensity is illustrated on the right side, *n* = 5/group. Data are presented as mean ± SD. Statistical analysis was conducted using a Two-tailed Student's T-test. **P* < 0.05, ***P* < 0.01, ****P* < 0.001, *****P* < 0.0001.

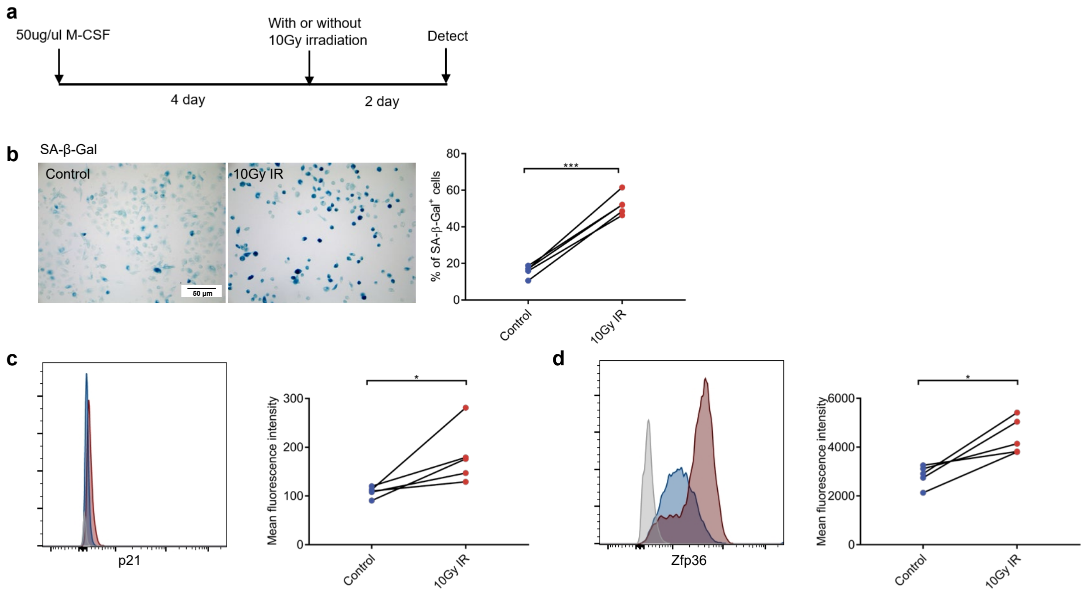

Supplementary Fig. 6 p21 and Zfp36 were upregulated in irradiation-induced senescent BMDMs. a Schematic illustration of irradiation-induced senescent BMDMs. **b** Representative image of SA-β-Gal staining of BMDMs with or without irradiation. Scale bar, 50 µm. Quantitative analysis of SA-β-Gal $^{+}$ cells is illustrated on the right side, *n* = 5/group. **c** Representative flow panels of p21 expression in BMDMs. Iso control was shown in grey. Quantitative analysis of mean fluorescence intensity is illustrated on the right side, *n* = 5/group. **d** Representative flow panels of Zfp36 expression in BMDMs. Iso control was shown in grey. Quantitative analysis of mean fluorescence intensity is illustrated on the right side, *n* = 5/group. Data are presented as mean ± SD. Statistical analysis was conducted using Two-tailed Student's T-test (b-d). *$P < 0.05$, ***$P < 0.001$.

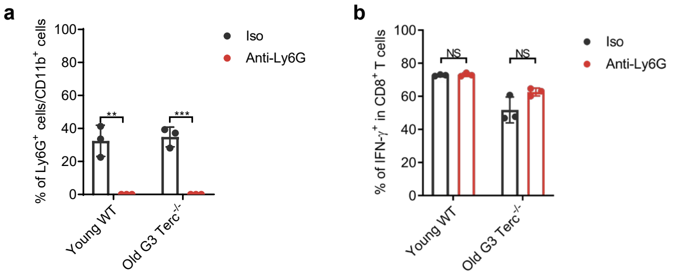

Supplementary Fig. 7 Neutrophil depletion had little effect on IFN-γ^{+}CD8^{+} T ratio in allografts. a Neutrophils in Young WT and Old G3 Terc$^{-/-}$ mice allografts treated with Iso or Anti-Ly6G. **b** IFN-γ^{+}CD8^{+} T cells from allografts of Young WT and Old G3 Terc$^{-/-}$ mice treated with Iso or Anti-Ly6G, *n* = 3/group. **$P < 0.01$, ***$P < 0.001$.

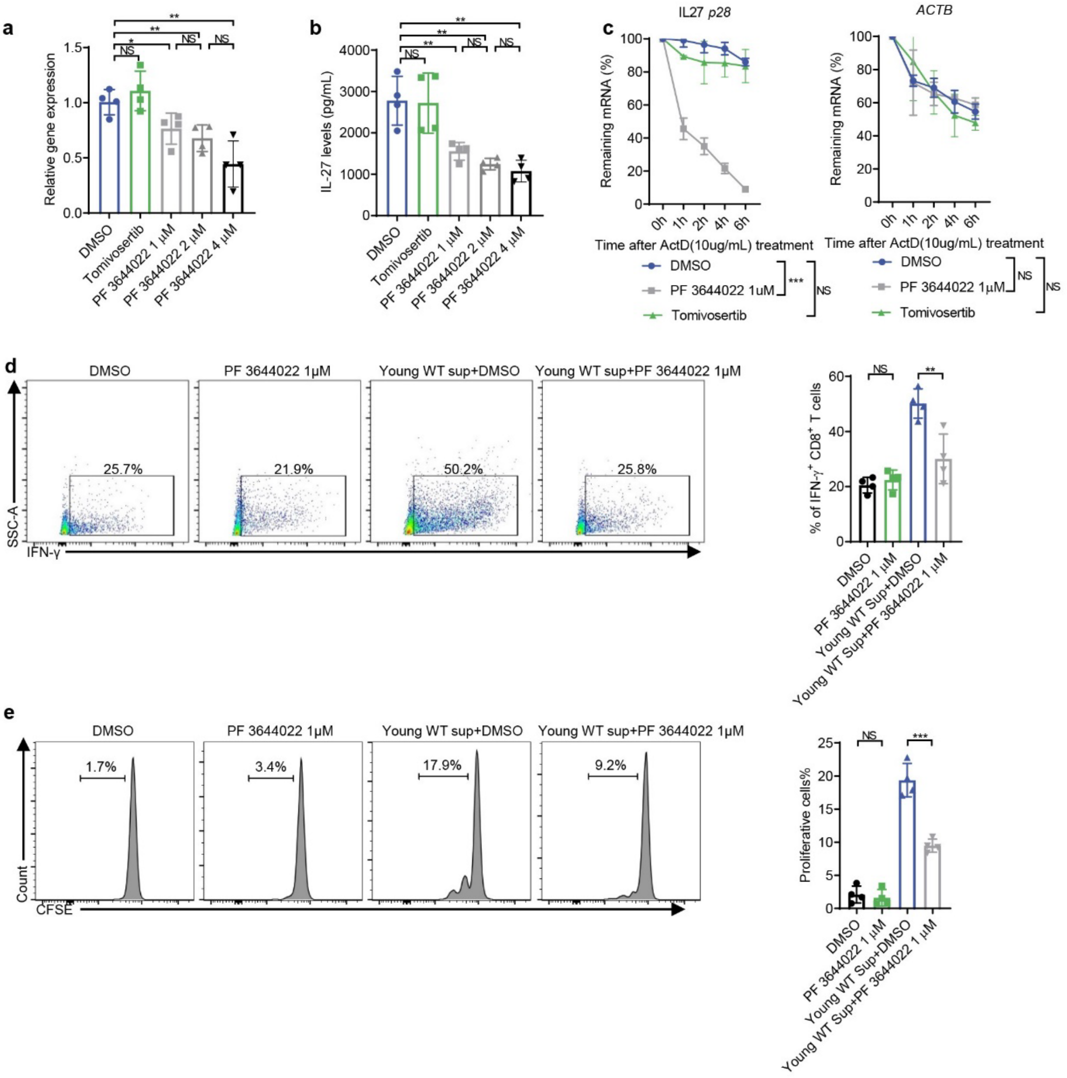

Supplementary Fig. 8 MK2 inhibitor PF 3644022 reduced IL-27 production of young BMDMs and promoted CD8$^+$ T cell activation and proliferation. a Expression levels of BMDM IL-27 *p28* in response to various concentrations of PF 3644022 analyzed using qPCR. Tomivosertib served as an irrelevant kinase inhibitor, n = 4/group. **b** IL-27 levels in BMDM supernatants after treatment with varying concentrations of PF 3644022 by ELISA, n = 4/group. **c** IL-27 *p28* and *ACTB* mRNA degradation of DMSO- or PF 43644022-treated BMDMs analyzed by qPCR after blocking RNA *de novo* synthesis using ActD, n = 4/group. **d** Naïve CD8$^+$ T cells were cultured with DMSO or PF 43644022-treated BMDM supernatant. Representative flow panels of IFN-γ^+CD8$^+$ T cells. Quantitative analysis of the IFN-γ^+CD8$^+$ T ratios is illustrated on the right side, n = 4/group. **e** Representative flow panels of CFSE-labeled CD8$^+$ T cells. Quantitative analysis of proliferative CD8$^+$ T is illustrated on the right side, n = 4/group. Data are presented as mean ± SD. Statistical analysis was performed using Two-tailed Student's T-test. *$P < 0.05$, **$P < 0.01$, ***$P < 0.001$, ****$P < 0.0001$, NS, not significant.

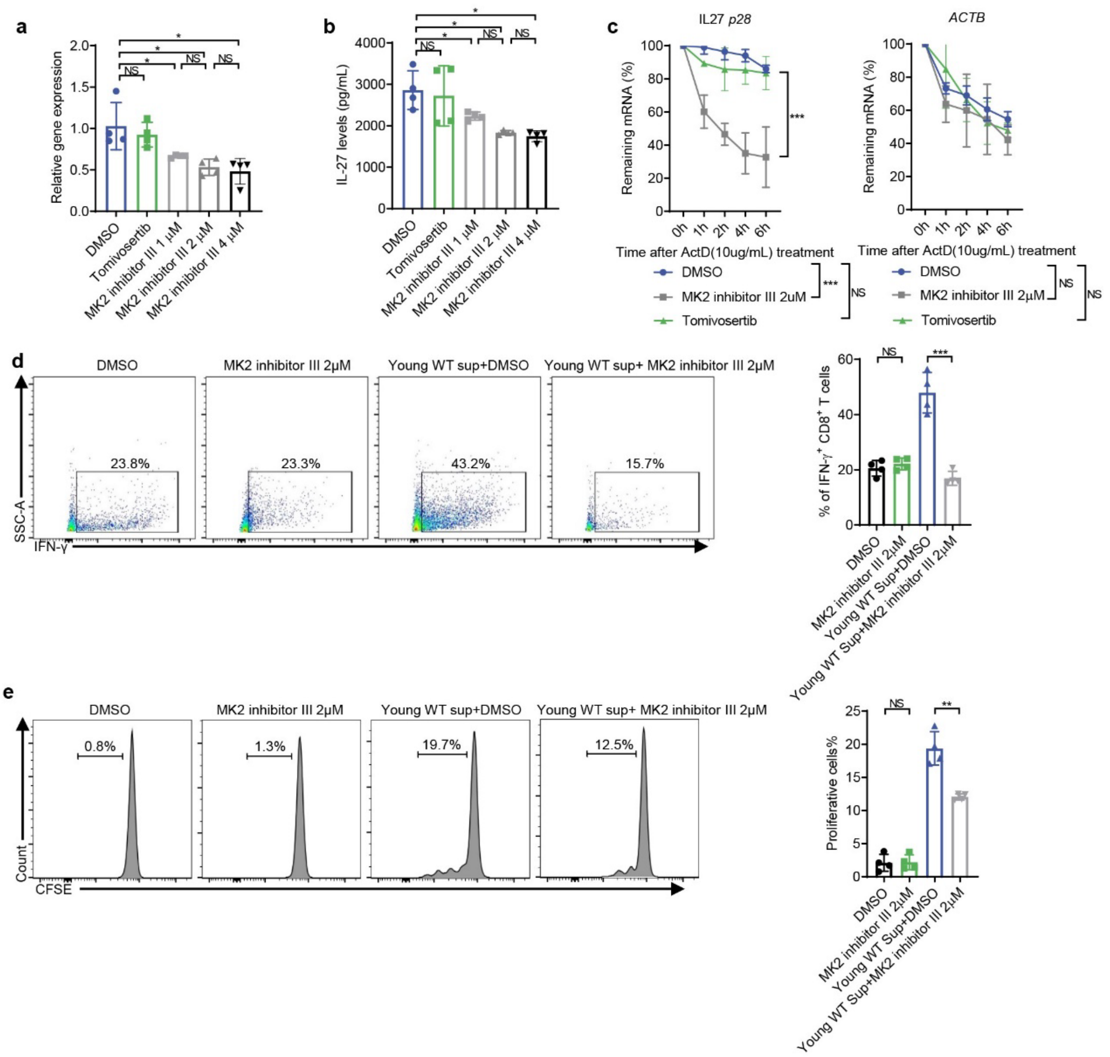

Supplementary Fig. 9 MK2 inhibitor III reduced IL-27 production of young BMDMs and promoted CD8⁺ T cell activation and proliferation. a Expression level of BMDM IL-27 *p28* in response to different concentrations of MK2 inhibitor III analyzed through qPCR, Tomivosertib served as an irrelevant kinase inhibitor, n = 4/group. **b** IL27 levels in BMDM supernatants after exposure to different concentrations of MK2 inhibitor III by ELISA, n = 4/group. **c** IL-27 *p28* and *ACTB* mRNA degradation of DMSO- or MK2 inhibitor III-treated BMDMs analyzed using qPCR after blocking RNA *de novo* synthesis using ActD, n = 4/group. **d** Naïve $CD8^+$ T cells were cultured with DMSO or MK2 inhibitor III-treated BMDM supernatant. Representative flow panels of IFN-γ^+CD8^+ T cells. Quantitative analysis of the IFN-γ^+CD8^+ T ratios is illustrated on the right side, n = 4/group. **e** Representative flow panels of CFSE-labeled $CD8^+$ T cells. Quantitative analysis of the proliferative $CD8^+$ T is illustrated on the right side, n = 4/group. Data are presented as mean ± SD. Statistical analysis was performed using Two-tailed Student's T-test. *$P < 0.05$, **$P < 0.01$, ***$P < 0.001$, ****$P < 0.0001$, NS, not significant.

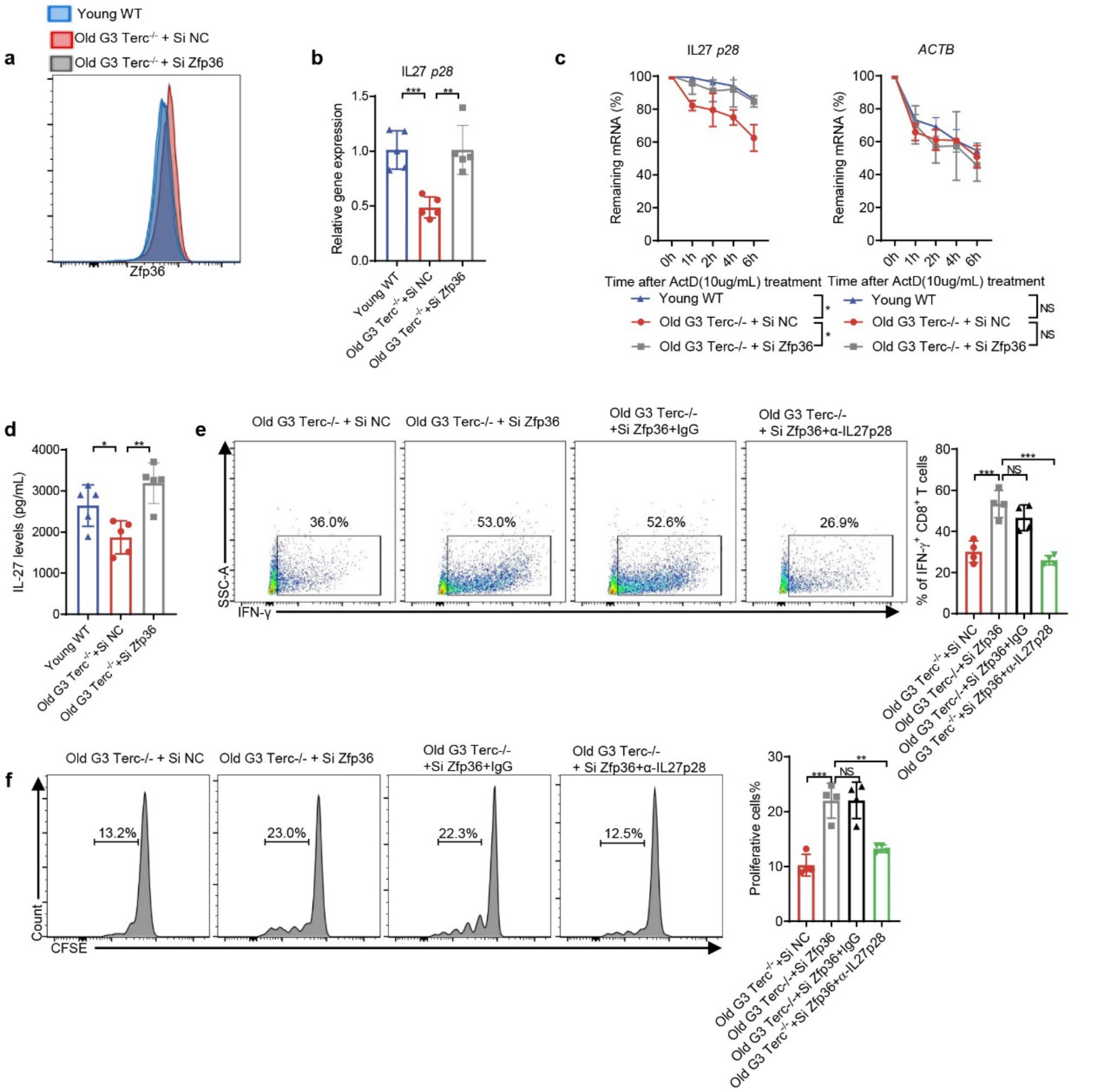

Supplementary Fig. 10 Knockdown of Zfp36 in senescent BMDMs restored IL-27 production and promotion of CD8$^+$ T cell activation and proliferation, which was counteracted by neutralization IL-27. a Representative flow panels of Zfp36 expression in Young WT, Old G3 Terc$^{-/-}$ BMDMs treated with Si NC and Si Zfp36. **b** Expression levels of BMDM IL-27 *p28* were analyzed using qPCR, *n* = 5/group. **c** IL-27 *p28* and *ACTB* mRNA degradation analyzed using qPCR after blocking RNA *de novo* synthesis using ActD, *n* = 5/group. **d** IL-27 levels in BMDM supernatants analyzed using ELISA, *n* = 5/group. **e** Naïve CD8$^+$ T cells were cultured with Si NC- or Si Zfp36-treated and Si Zfp36-treated with IgG or α-IL27p28 BMDM supernatant. Representative flow panels of IFN-γ$^+$CD8$^+$ T cells. Quantitative analysis of the IFN-γ$^+$CD8$^+$ T ratios is illustrated on the right side, *n* = 5/group. **f** Representative flow panels of CFSE-labeled CD8$^+$ T cells. Quantitative analysis of the proliferative CD8$^+$ T is illustrated on the right side, *n* = 5/group. Data are presented as mean ± SD. Statistical analysis was performed using Two-tailed Student's T-test (b-f). **P* < 0.05, ***P* < 0.01, ****P* < 0.001, NS, not significant.

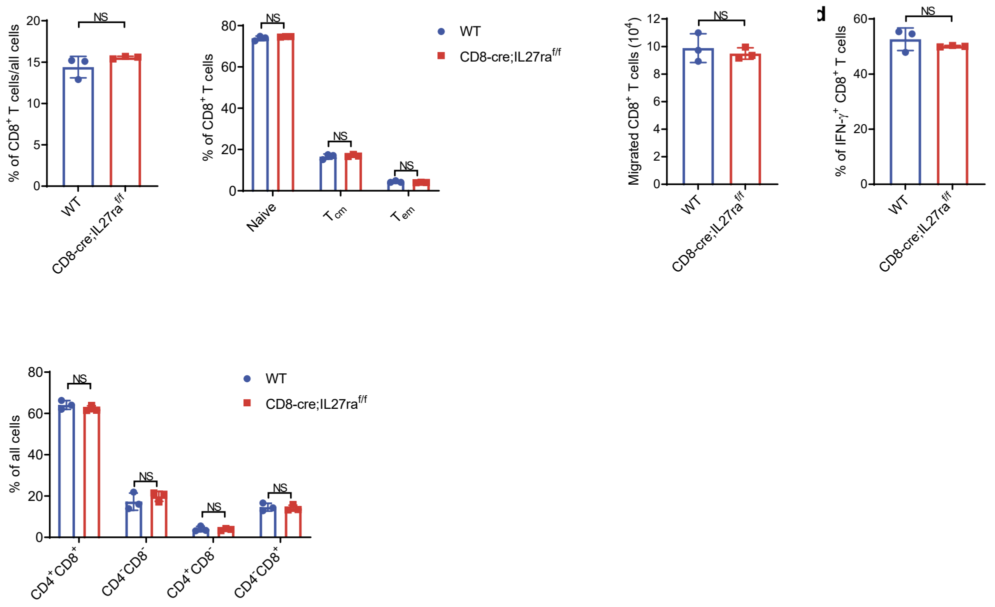

Supplementary Fig. 11 Cd8-cre; IL27Ra$^{f/f}$ cause no other CD8 T cell defects in numbers, migration, maturation, and homeostasis. a Proportion of CD8$^+$ T cells in spleen cells. **b** Proportion of Naïve CD8$^+$ T cell, T_{cm} and T_{em} in spleen CD8$^+$ T cells. **c** Migration CD8$^+$ T cells numbers in lower chamber of Transwell® plate. **d** IFN-γ$^+$CD8$^+$ T ratios after Naïve CD8$^+$ T cell was activated for 72h. **e** Proportion of CD4$^+$CD8$^+$cells, CD4$^-$CD8$^-$cells, CD4$^+$CD8$^-$cells, CD4$^-$CD8$^+$cells in thymus cells, *n* = 3/group. Data are presented as mean ± SD. Statistical analysis was performed using Two-tailed Student's T-test. NS, not significant.

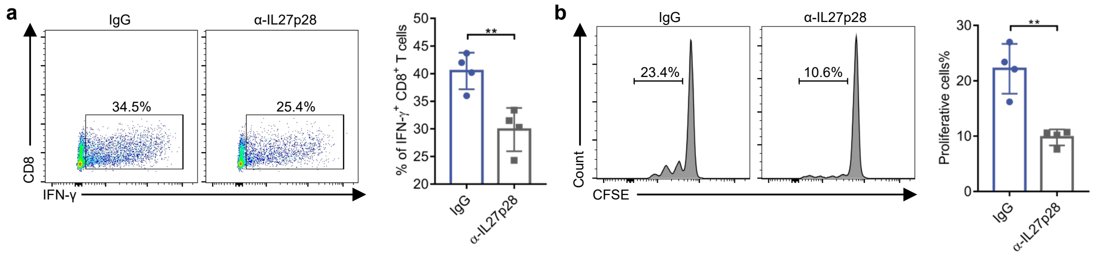

Supplementary Fig. 12 Neutralization IL-27 of young macrophage supernatant attenuated the promotion of CD8$^+$ T cell activation and proliferation. a Naïve CD8$^+$ T cells were cultured alongside Young WT BMDM supernatant with IgG or α-IL27p28. Representative flow panels of IFN-γ$^+$CD8$^+$ T cells. Quantitative analysis of the IFN-γ$^+$CD8$^+$ T ratios is illustrated on the right side, *n* = 4/group. **b** Representative flow panels of CFSE-labeled CD8$^+$ T cells. Quantitative analysis of the proliferative CD8$^+$ T is illustrated on the right side, *n* = 4/group. Data are presented as mean ± SD. Statistical analysis was performed using Two-tailed Student's T-test. $^{**}P < 0.01$.

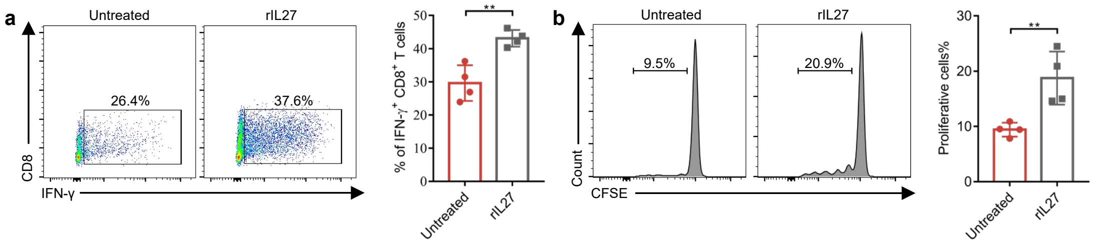

Supplementary Fig. 13 Supplementation of IL-27 to senescent macrophage supernatant enhanced promotion of $CD8^+$ T cell activation and proliferation. a Naïve $CD8^+$ T cells were cultured with Old G3 $Terc^{-/-}$ BMDM supernatant with PBS or rIL27. Representative flow panels of IFN-γ^+CD8^+ T cells. Quantitative analysis of IFN-γ^+CD8^+ T ratios is illustrated on the right side, n = 4/group. **b** Representative flow panels of CFSE-labeled $CD8^+$ T cells. Quantitative analysis of the proliferative $CD8^+$ T is illustrated on the right side, n = 4/group. Data are presented as mean ± SD. Statistical analysis was performed using Two-tailed Student's T-test. $^{**}P < 0.01$.

Supplementary Table 1 Demographics and baseline characteristics of recipients and donors, donor-recipient matching information and immunosuppression therapy.

**Characteristic**	**ACR** (*n* = 119)	**Non ACR** (*n* = 119)	***P*-value**
**Recipients**			
Age (year, Mean±SD)	41.68±11.10	39.81±9.84	0.154
Height (cm, Mean±SD)	165.16±8.10	165.35±8.03	0.853
Weight (kg, Mean±SD)	59.37±11.45	60.10±13.01	0.632
BMI (kg/m^2, Mean ± SD)	21.63±3.03	21.86±3.86	0.980
BSA (m^2, Mean ± SD)	1.65±0.19	1.65±0.20	0.709
Primary cause of ESKD (n, %)			0.558
Glomerular disease	96 (80.07%)	103 (86.55%)	
IgA nephropathy	1 (0.84%)	0 (0%)	
lupus nephritis	1 (0.84%)	1 (0.84%)	
Polycystic kidney disease	5 (4.58%)	5 (4.58%)	
Diabetic Nephropathy	3 (2.52%)	4 (3.36%)	
Hypertensive nephrosclerosis	3 (2.52%)	3 (2.52%)	
Obstructive nephropathy	1 (0.84%)	1 (0.84%)	
Other	9 (8.26%)	2 (1.68%)	
History of dialysis (n, %)			
Hemodialysis	73 (61.34%)	72 (60.50%)	0.602
Peritoneal dialysis	39 (32.77%)	36 (30.25%)	
None	7 (5.88%)	11 (9.24%)	
Time on dialysis prior to transplantation（days, IQR）	1608 (803-2265)	1104 (87-2209)	**0.018**
**Donors**			
Age (year, Mean ± SD)	41.88 ±11.26	47.84 ±11.32	**<0.001**
Gender (M, %)	75 (63.0%)	66 (55.5%)	0.235
Height (cm, Mean ± SD)	165.58±7.49	162.62±8.22	**0.001**

Weight (kg, Mean ± SD)	59.30±13.02	59.68±10.77	0.544
BMI (Mean ± SD)	21.53±3.93	22.55±3.65	**0.014**
BSA (m^2, Mean ± SD)	1.64±0.20	1.64±0.17	0.700
**Donor-recipient matching information**			
Donor-recipient BMI ratio（Mean ± SD）	1.02±0.23	1.07±0.25	**0.026**
Donor-recipient BSA ratio（Mean ± SD）	1.04±0.17	1.03±0.16	0.739
HLA mismatches (n, %)			0.071
0	8 (6.72%)	12 (10.08%)	
1	6 (5.04%)	6 (5.04%)	
2	25 (21.01%)	31 (26.05%)	
3	35 (29.41%)	36 (30.25%)	
4	34 (28.57%)	17 (14.29%)	
5	9 (7.56%)	8 (5.88%)	
6	2 (1.68%)	9 (8.40%)	
**Immunosuppression therapy**			
FK506+Pre+MMF	102(85.71%)	106 (89.08%)	0.500
CsA+Pre+MMF	16 (13.45%)	13 (10.92%)	
Other	1 (0.84%)	0 (0%)	

ACR, acute cellular rejection; Non ACR, non-acute cellular rejection; BMI, body mass index; BSA, body surface area; DCD, donation after cardiac death; DBD, donation after brain death. Bold denotes statistical difference. Pre, prednisolone. MMF: Mycophenolate mofetil. CsA: Ciclosporin A.

Supplementary Table2 Banff type of rejection

**Banff type**	**ACR (*n* = 119)**
IA	58 (48.74%)
IIA	21 (17.64%)
IB	28 (23.53%)
IIB	4 (3.36%)
IIA+IB	5 (4.20%)
IA+IIB	2 (1.68%)
III	1 (0.84)

Supplementary Table 3 Sequence of AU-rich element ATTTA pentamer in mouse IL-27 *p28* 3'UTR

Chromosome	Start	End	Motif
chr7	126589067	126589072	ATTTA
chr7	126589089	126589094	ATTTA
chr7	126589093	126589098	ATTTA
chr7	126589108	126589113	ATTTA
chr7	126589137	126589142	ATTTA

Supplementary Table 4 Sequence of AU-rich element ATTTA pentamer in human IL-27 *p28* 3'UTR

Chromosome	Start	End	Motif
Chr16	28499433	28499438	ATTTA
Chr16	28499460	28499465	ATTTA
Chr16	28499464	28499469	ATTTA
Chr16	28499479	28499484	ATTTA

Supplementary Table 5 Primer sequences for Telomere length measurements

Primer	Sequences (5’-3’)
Tel F	CGGTTTGTTTGGGTTTGGGTTTGGGTTTGGGTTTGGGTT
Tel R	GGCTTGCCTTACCCTTACCCTTACCCTTACCCTTACCCT
β-globin F	GCTTCTGACACAACTGTGTTCACTAGC
β-globin R	CACCAACTTCATCCACGTTCACC

Supplementary Table 6 Primer sequences for RT-qPCR

Primer	Sequences (5'-3')
Gapdh F	TCATCCCAGAGCTGAACG
Gapdh R	TCATACTTGGCAGGTTTCTCC
Ifn-g F	CGGCACAGTCATTGAAAGCC
Ifn-g R	TGCATCCTTTTTCGCCTTGC
Gzmb F	GAAGCCAGGAGATGTGTGCT
Gzmb R	GCACGTTTGGTCTTTGGGTC
Prf1 F	TCTTGGTGGGACTTCAGCTT
Prf1 R	TGCTTGCATTCTGACCGAGT
Tbx21 F	ATTGGTTGGAGAGGAAGCGG
Tbx21 R	GAACCATGGGGTCAGAAGCA
ATP1a1 F	CACGTGGCAGCCCTAGTTC
ATP1a1 R	ATACAGCGGCAGGCTCATAC
Miox F	TCCTGGGGCCATGATGAGTA
Miox R	CCTTGATAGTAGGGCCGCAG
S100A6 F	GCCCGCTAAACTCCCTCATT
S100A6 R	GTGGAAGATGGCCACGAGAA
p28 F	CTCTGCTTCCTCGCTACCAC
p28 R	GGGGCAGCTTCTTTTCTTCT
